# Transmetalation From Boron to Beryllium in Phosphorus‐Based Scorpionate Complexes

**DOI:** 10.1002/chem.202500673

**Published:** 2025-03-30

**Authors:** Chantsalmaa Berthold, Mark H. Lochte, Magnus R. Buchner

**Affiliations:** ^1^ Fachbereich Chemie Philipps‐Universität Marburg 35043 Marburg Germany

**Keywords:** beryllium, boron, cross coupling, main group chemistry, transmetalation

## Abstract

Investigation of tris(di‐iso‐propylphosphanylmethyl)phenylborate ([TP(*i*Pr)]^−^) organo‐beryllium complexes [TP(*i*Pr)]Be*R* with *R* = Ph, *n*Bu, Cp, Cp* revealed transmetalation of [CH_2_P(*i*Pr)_2_]^−^ groups from boron onto beryllium. This reaction is caused by partial dissociation of the scorpionate, which can be triggered through steric overcrowding of the beryllium atom or reducing the ligand beryllium bond strength through oxidation of the phosphorus atoms with selenium. Oxidation with oxygen or sulfur results in the formation of stable phosphine oxide and sulfide scorpionates

## Graphical abstract

Transmetalation from boron onto beryllium was observed. This reaction is triggered through steric overcrowding of the beryllium atom or reducing the ligand beryllium bond strength through oxidation of the ligating phosphorus atoms.

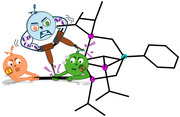

The interest in the lightest group 2 element has increased rapidly in the last 5 year.^[^ ^1^, ^2^ ^]^ However, these studies have predominantly focused on low‐valent beryllium complexes or beryllium metal bonds to other main group metals, while still little is known on the reactivity of beryllium compounds. In the last few years, our group has been able to show that beryllium compounds are closely related to boron species with similar ligand spheres.^[^ ^3^ ^]^ Continuing on in this vein, we serendipitously discovered the transfer of [CH_2_P(*i*Pr)_2_]^−^ groups from boron onto beryllium. This is, to the best of our knowledge, the first example of transmetalation from a more electronegative to a less electronegative element. Thus, this observation is important to shed light onto the still not fully understood transmetalation step in Suzuki‐type cross‐coupling reactions.^[^ ^4^ ^]^ In order to understand this behavior, we investigated the conditions leading to this carbon atom transfer from boron to beryllium.

Lithium [tris(di‐iso‐propylphosphanylmethyl)phenylborate] ([TP(*i*Pr)]Li(thf))^[^ ^5^, ^6^ ^]^ has successfully been employed for the synthesis of beryllium halide and pseudohalide complexes ([TP(*i*Pr)]Be*L* (*L*  = Cl, Br, I, CN, NCS, NCO, CF_3_SO_3_). In these complexes the ^1^ *J*_PBe_ NMR coupling constant is an excellent spectroscopic probe for the pK_b_ value of *L*.^[^ ^7^ ^]^ Therefore, we attempted to extend this work through the synthesis of [tris(di‐iso‐propylphosphanylmethyl)phenylborate] organo‐beryllium complexes ([TP(*i*Pr)]Be*R*), which are in analogy to TpBe*R* (Tp  =  1‑tris(pyrazolyl)borate; *R*  =  Ph, *n*Bu, Me, carbenes).^[^ ^8^ ^]^ Through evaluation of the ^1^ *J*_PBe_ NMR coupling constant in [TP(*i*Pr)]Be*R* we intended to evaluate the base strength of the neutral and anionic C‐ligands. At −50 °C the reaction of one equivalent of [TP(*i*Pr)]Li(thf) with one equivalent of beryllium Grignard compound ([(Et_2_O)Be*R*Cl] *R*  = Ph, *n*Bu)^[^ ^9^ ^]^ in toluene led to quantitative formation of organo‐beryllium complexes [TP(*i*Pr)]Be*R* (**1a**: *R*  = Ph, **1b**: R  = *n*Bu, Scheme 1a). Complexes **1a** and **1b** were investigated using multinuclear NMR und IR spectroscopy. Generally, at ambient temperature broad singlets were observed in the ^9^Be{^1^H} and ^31^P{^1^H} NMR spectra at 2.41 and −4.48 ppm for **1a** and at 2.36 and −2.81 ppm for **1b**, respectively. Due to coupling of three equivalent ^31^P nuclei with spin 1/2 to one ^9^Be nucleus with spin 3/2,^[^ ^10^ ^]^ characteristic quartets in the ^9^Be{^1^H} and ^31^P{^1^H} NMR spectra would be expected, in analogy to those found for the halide and pseudohalide complexes [TP(*i*Pr)]Be*L*.^[^ ^7^ ^]^ Though, even at temperatures as low as 223 K, coupling could not be resolved (Supporting Information Figures S10 and S15). However, the ^9^Be NMR signal of organic beryllium compounds tends to be very broad and this interaction seems to also broaden the ^1^ *J*_PBe_ coupling significantly.^[^ ^9^, ^11^ ^]^ The ^9^Be{^1^H} and ^31^P{^1^H} NMR signals of **1a** and **1b** are found at lower field compared to the chemical shifts of [TP(*i*Pr)]Be*L* (*L*  = Br, I, CN, NCS, NCO).^[^ ^7^ ^]^ In the ^9^Be{^1^H} NMR spectra the chemical shifts of **1a** and **1b** are shifted upfield in comparison to the neutral organoberyllium Tp complexes (TpBe*R*: 5.83–7.18 ppm), and the half‐width is significantly increased to 143.3 and 96.6 Hz (TpBe*R*: ω_1/2 _= 16–40.9 ppm).^[^ ^8^ ^]^ The ^1^H, ^13^C{^1^H}, and ^11^B{^1^H} NMR spectra show the expected signal sets (Supporting Information).

**Scheme 1:**
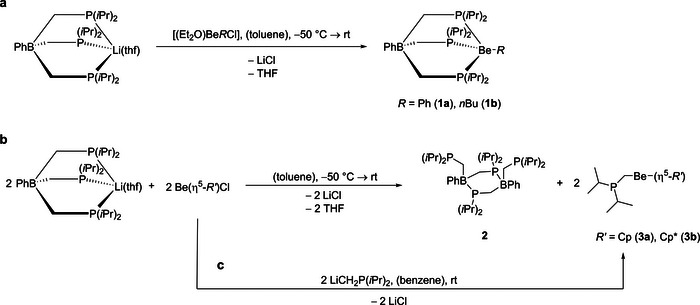
Synthesis of [TP(*i*Pr)]Be*R* (*R* = Ph (**1a**), *n*Bu (**1b**)) a), compound **2** b) and [Be(R′) (CH_2_P(*i*Pr)_2_)] (R′ = Cp (**3a**), Cp* (**3b**)) c).

Compound **1a** can be crystallized from concentrated toluene solution at −34 °C. Compound **1a** crystallizes in the orthorhombic space group P2_1_2_1_2_1_ with four formula units per unit cell (Supporting Information Table S1). The beryllium atom is pseudo‐tetrahedrally coordinated by three phosphorous atoms and by one carbon atom, which is part of the phenyl ligand (Figure 1). At 1.7655(2) Å the Be─C atomic distance is longer than in the neutral organo‐beryllium complexes TpBe*R* (1.71–1.74 Å) but significantly shorter than in the cationic organo‐beryllium complexes [TpBe*R*]^+^ (1.80–1.82 Å).^[^ ^8^ ^]^ The Be─P bond lengths (2.2252(1)–2.2642(4) Å) in **1a** are significantly longer in comparison to other [TP(*i*Pr)]Be*L* (*L*  = Cl, Br, I, CN, NCS, NCO) complexes (2.1717(0)–2.2206(8) Å).^[^ ^7^ ^]^ The extended bond lengths can probably be attributed to steric repulsion due to the presence of bulky iso‐propyl groups at the phosphorus atom.

**Figure 1:**
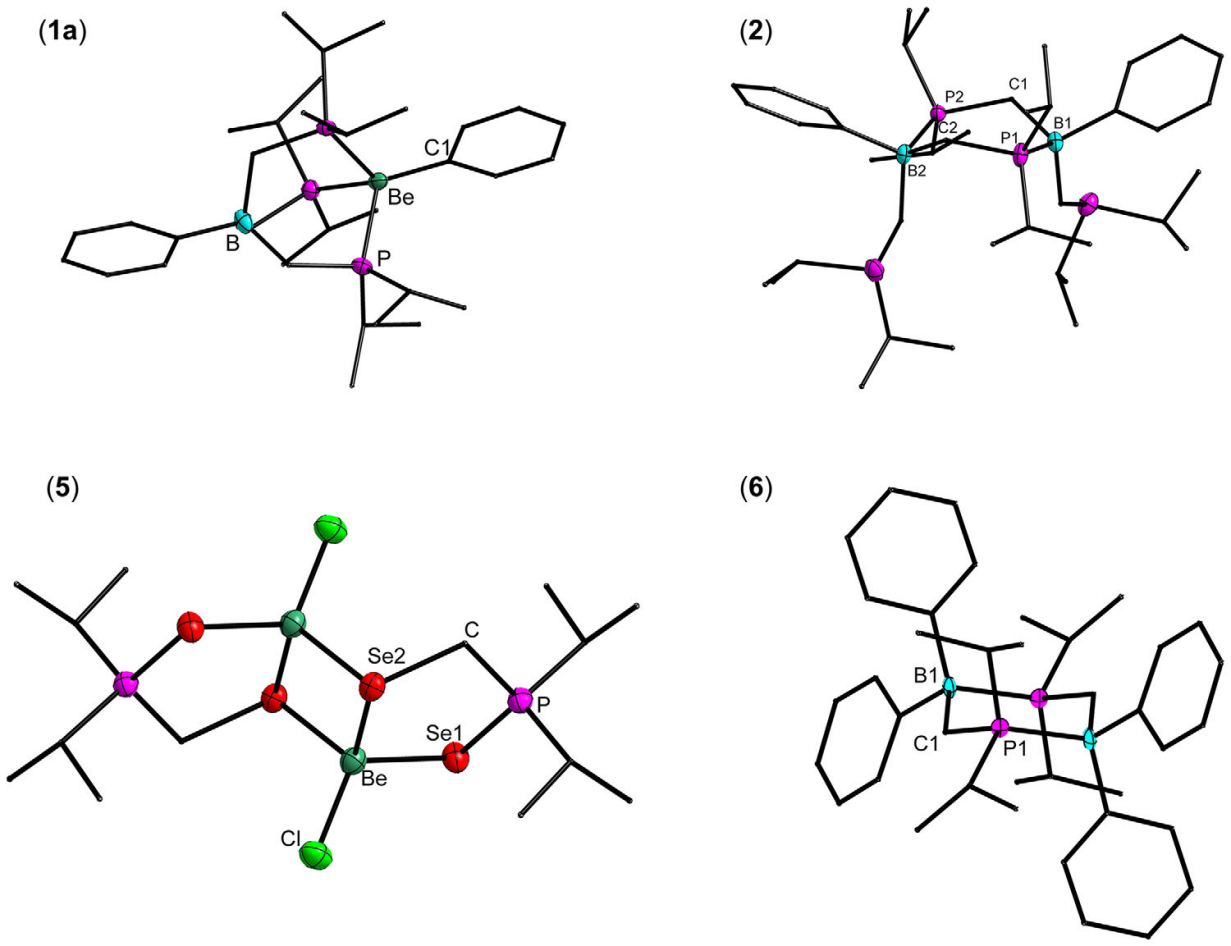
Molecular structures of **1a**, **2**, **5**, and **6** in the solid state. The ellipsoids are shown at 70% probability at 100 K. Hydrogen atoms are omitted, and carbon atoms are shown as wireframe for clarity.

The attempt to isolated [TP(*i*Pr)]Be*R*′ complexes with cyclopentadienyl (Cp) or pentamethylcyclopentadienyl (Cp*) ligands from the reaction of the respective beryllium half‐sandwich complexes Be*R*′Cl (*R*′  = Cp,^[^ ^12^ ^]^ Cp*^[^ ^13^ ^]^) and [TP(*i*Pr)]Li(thf) was unsuccessful (Figure 1b). [TP(*i*Pr)]BeCl was observed in the *in situ* ^9^Be{^1^H} NMR spectrum, along with a new kind of Be‐Cp/Cp* species in the high‐field region at −19 to −16 ppm (Supporting Information Figure S63). However, we were expecting a downfield shift in the ^9^Be{^1^H} NMR spectra, similarly to the tris(pyrazolyl)borate complexes TpBe(η^1^‐*R*′) (*R*′  = Cp, Cp*) compounds. The *in situ* ^31^P{^1^H} NMR spectra showed the formation of many unidentified products. We were able to identify the species liable for the signals with highest intensity. The signals found at 9.31 and 3.17 ppm are assigned to the dinuclear boron species [PhB{CH_2_P(*i*Pr)_2_}μ‐P(*i*Pr)_2_CH_2_]_2_ (**2**) and the signals at 3.30 and 9.04 ppm are of [Be(η^5^‐Cp) (CH_2_P(*i*Pr)_2_)] (**3a**) and [Be(η^1^‐Cp*) (CH_2_P(*i*Pr)_2_)] (**3b**), respectively. The identity of **2** was additionally verified by X‐ray diffraction analysis (Figure 1, see also below). For additional characterization of **2** via NMR and IR spectroscopy see the Supporting Information. Compounds **3a** and **3b** were selectively synthesized by salt elimination from di‐iso‐propylphosphanylmethyllithium (LiCH_2_P(*i*Pr_2_)^[^ ^6^ ^]^) and Be*R*′Cl (Figure 1c). The ^9^Be{^1^H} and ^31^P{^1^H} NMR spectra of **3a** and **3b** show broad singlets at −19.6 (ω_1/2_  = 23.3 Hz) and 9.04 ppm as well as −17.0 (ω_1/2_  =  21.9 Hz) and 5.30 ppm, respectively. ^1^H and ^13^C{^1^H} NMR as well as IR spectroscopy additionally confirm the identity of **3a** and **3b** (see Supporting Information). While in TpBe(η^1^‐R′) (R′  = Cp, Cp*) the Be─N bond is so strong that no ligand dissociation occurs, in the TP systems, the Be─P bond is weak enough, that η^3^ coordination of the cyclopentadienyl ligands is feasible. This coordination mode has already been observed to induce increased reactivity compared to η^5^ coordination.^[^ ^1^ ^]^ Therefore, this increased reactivity in combination with the high steric pressure induced by the isopropyl groups together with the cyclopentadienyl ligands enforces [CH_2_P(*i*Pr)_2_]^−^ group transfer from boron onto beryllium.

Considering the good correlation of the ^1^ *J*_PBe_ NMR coupling constant with the pK_b_ value of the coordinated base in the halide and pseudohalide complexes ([TP(*i*Pr)]Be*L* (*L*  = Cl, Br, I, CN, NCS, NCO, CF_3_SO_3_), we attempted to extend this system to neutral Lewis bases, in analogy to the Tp compounds.^[^ ^7^, ^8^ ^]^ However, all attempts to abstract the anionic ligand from the beryllium center while providing a neutral donor ligand resulted in the decomposition of the phosphanylborate. Some of the decomposition products could be elucidated, such as compound **2**. We assumed this decomposition is caused by the relatively weak Be─P bond in combination with the increased Lewis acidity of the beryllium atom upon generation of cationic intermediates. Therefore, we conducted studies on the influence of the Be─scorpionate bond strength upon the reactivity of these complexes through insertion of a group 16 atom into the Be─P bond (Scheme 2). We focused exclusively on the chloride and partially on the phenyl compound [TP(*i*Pr)]BeCl/Ph, due to the fact that the Be─P bond in these systems was well understood from previous studies, allowing us to infer the reactivity of related compounds.

**Scheme 2:**
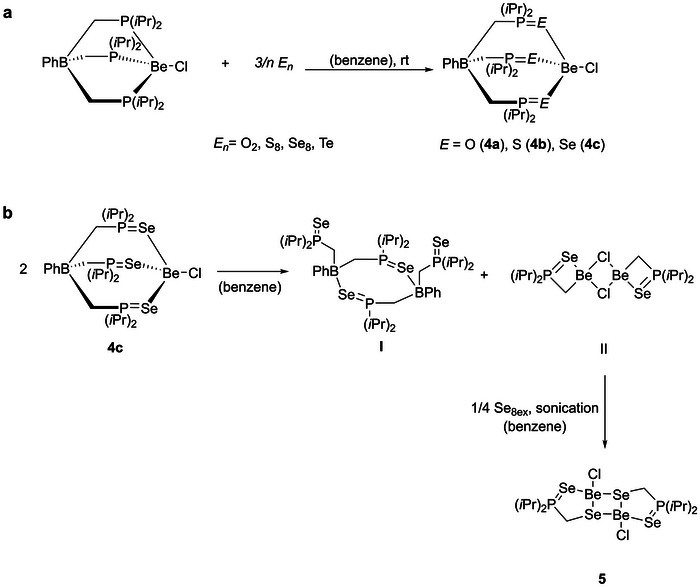
Synthetic routes toward [TP(*i*Pr)E]BeCl (E = O (**4a**), S (**4b**), Se (**4c**)), and compound **5**.

[TP(*i*Pr)]BeCl or **1a** were dissolved in benzene. After several freeze‐pump‐thaw degassing cycles, the mixture was exposed to 2 bars of oxygen at ambient temperature. Reactions with sulfur and selenium were carried out under similar conditions by mixing the starting materials in the solid state followed by suspension in benzene. The ^31^P{^1^H} and ^9^Be{^1^H} NMR signals of [TP(*i*Pr)O]BePh, [TP(*i*Pr)O]BeCl, [TP(*i*Pr)S]BeCl, and [TP(iPr)Se]BeCl were observed at 73.5 and 0.89 ppm, 75.0 and 1.79 ppm, 64.0 and 6.08 ppm as well as 50.3 ppm (^1^ *J*_SeP_ = 651 Hz) and 4.83 ppm, respectively. In the ^77^Se NMR spectrum of **4c** a doublet at −357 ppm (^1^ *J*_SeP_ = 651 Hz) was observed. **4a**–**d** show one sharp signal in their ^11^B{^1^H} NMR spectra within a small spectral window (−13.3 to −15.4 ppm). While monitoring the reaction with selenium by ^9^Be{^1^H} and ^31^P{^1^H} NMR spectroscopy, cleavage of **4c** in the presence of excess selenium was observed (Supporting Information Figures S51 and S52). Initially, two signals were detected in the ^9^Be{^1^H} NMR spectrum (Supporting Information Figure S51). The quartet with a chemical shift of 2.91 ppm is assigned to the starting material [TP(*i*Pr)]BeCl and a broad singlet at 4.83 ppm with a half‐width of 6.9 Hz is of species **4c**. After 24 hours of ultrasonic treatment, a downfield shifted singlet with a half‐width of 33.6 Hz was observed at 7.40 ppm, and the intensity of **4c** was significantly reduced. The complex [(Cl)Be{κ–Se(*i*Pr)_2_PCH_2_}μ–Se]_2_ (**5**) is attributed to this ^9^Be{^1^H} NMR signal. Single crystals of **5** were obtained from toluene at −34 °C (Figure 1). Compound **5** crystallizes in the monoclinic space group P2_1_/n with two formula units per unit cell. The beryllium atom is tetrahedrally coordinated by three selenium atoms and one chlorine atom. Two of these selenium atoms are crystallographically identical. The chlorine atom is terminal. The Se_μ_2 atom is the bridging element in this dimeric complex. The Se_t_1 atom forms a five‐membered ring with the CH_2_P(*i*Pr)_2_ moiety and the Se2 and Be atoms. The bond length between the Be and Cl atoms is 1.946(4) Å, which agrees with distances found for other beryllium chloride complexes^[^ ^14^ ^]^ and with the Be─Cl atomic distances from [TP(*i*Pr)]BeCl. The Be─Se distance to the bridging Se_μ_2 atom is 2.274(4) Å, while the Se_t_1─Be separation is significantly shorter at 2.257(4) Å. A previously determined Be─Se atomic distance of 2.179(3) Å is significantly shorter.^[^ ^15^ ^]^ The distance between the Se_t_1 and the P atom is 2.153(8) Å, which is smaller than distances between the Se and P atoms (2.233(4) Å) previously reported.^[^ ^16^ ^]^

We propose that the formation of **5** from **4c** occurs due to an excess of selenium as depicted in Scheme 2b. First, transmetalation leads to the formation of neutral dinuclear boron species **I** and dinuclear beryllium complex **II**. The formation of **I** is plausible, since similar phosphorus species such as compound **2** have been observed repeatedly during thermal treatment or reactions with other reagents (Figure 1b). Complex **II** is in line with the formation of **3a** and **3b**, and the μ‐chloro‐bridged motif is abundant in beryllium chemistry.^[^ ^9^, ^17^ ^]^ **II** reacts with excess selenium to give **5**, due to the release of ring strain. Two new signals with ^77^Se satellites were observed at 61.3 and 50.2 ppm (Supporting Information Figures S51 and 52) in the ^31^P{^1^H} NMR spectrum of the reaction mixture. Considering ^31^P NMR signals are shifted downfield when the corresponding ^31^P nuclei are part of a five‐membered ring system,^[^ ^18^ ^]^ we assign the signal at 60.3 ppm to compound **5** and the signal at 50.2 ppm to byproduct **I**. For this compound a broad singlet in the ^11^B{^1^H} NMR spectrum at −12.3 ppm was observed. However, no ^77^Se{^1^H} signal of **5** or **I** could be observed.

No reaction occurred with tellurium under conditions similar to those applied for the other chalcogens. While heating to 100 °C overnight led to the formation of phosphanylborate [Ph_2_B(μ‐P(*i*Pr)_2_CH_2_)]_2_ (**6**), which we identified via X‐ray diffraction and NMR spectroscopy (Figure 1; see also the Supporting Information). The ^11^B{^1^H} and ^31^P{^1^H} NMR spectra of **6** feature one broad signal at −10.6 and 7.46 ppm, respectively. Compound **2** is structurally related to **6** (see above). The single crystals of **2** crystallize in the monoclinic space group C2_1_/c with eight formula units per unit cell, while **6** crystallizes in the monoclinic space group P2_1_/c with two formula units per unit cell. Both compounds are dinuclear in the solid state and the boron atoms are bridged by C─P moieties building a six‐membered ring like in cyclohexane. The molecular structure of **6** is close to the chair conformation with P1─B1─C1 and B1─ C1─P1 angles at 105.1(3)° and 108.86(2)°, which is the thermodynamically more stable conformation (111.1°).^[^ ^19^ ^]^ Compound **2** exhibits a twist boat conformation, the second energetic minimum. At 104.9(1)–105.0(1)° and 123.0(1–123.9(1)° the C1─B1─P1 and B2─C2─P2 angles are smaller than those described for related compounds (111.8, 116.6°).^[^ ^20^, ^21^ ^]^ The respective C─P, B─P, and B─C atomic distances of compounds **2** and **6** are in the range of 1.8184(8–1.8640(7), 2.0188(0)–2.0408(2) and 1.6261(4)–1.6635(4) Å, which fit very well with literature values.^[^ ^20^, ^21^ ^]^ Compound **6** is presumably also formed through [CH_2_P(*i*Pr)_2_]^−^ group transfer onto beryllium as well as Lewis acid promoted ligand scrambling at the boron atoms.

Furthermore, we investigated the reactivity of the Be─P bond toward the small molecules carbon monoxide, carbon dioxide, sulfur dioxide, and carbon disulfide (Supporting Information Figure S74). However, no selective reactivity was observed and many products, such as P─P species, oxidized phosphanylborates, and compound **2** were formed.

In summary, transmetalation from the more electronegative element boron to the less electronegative element beryllium is feasible. This carbon atom transfer is induced through strong steric pressure at the small beryllium atom, increased Lewis acidity of the beryllium atom, or due to a very weak beryllium‐scorpionate bond. This observed reactivity not only opens new avenues for the synthesis of unprecedented organo‐beryllium complexes but also delivers novel insight into the fundamental driving force of boron mediated transmetalation reactions.

## Experimental Section

Caution! Beryllium and its compounds are categorized as human carcinogens and work with these substances is associated with severe health hazards.^[^ ^22^ ^]^ As the biochemical mechanisms that cause beryllium‐associated diseases are still unknown,^[^ ^23^ ^]^ special (safety) precautions are strongly advised.^[^ ^22^ ^]^

Details on the synthesis, experimental setup, and characterization of the described compounds are given in the Supporting Information. The authors have cited additional references within the Supporting Information.^[^ ^24^ ^]^ Deposition Number(s) 2424804‐2424807 contain(s) the supplementary crystallographic data for this paper. These data are provided free of charge by the joint Cambridge Crystallographic Data Centre and Fachinformationszentrum Karlsruhe Access Structures service.

## Conflict of Interests

The authors declare no conflict of interest.

## Supporting information



Supporting Information
