# Supplementary material for: Transmetalation From Boron to Beryllium in Phosphorus‐Based Scorpionate Complexes
Source: Chemistry. 2025 Mar 30;31(24):e202500673. doi: 10.1002/chem.202500673 (PMC12043030; doi:10.1002/chem.202500673)
Supplement: Supplementary file 1 — Supporting Information [file CHEM-31-e202500673-s001.pdf]

Experimental Details 4
General Experimental Techniques 4
NMR spectroscopy 4
Single Crystal X-Ray Diffraction 4
IR Spectroscopy 5
Experimental Procedures 6
Preparation of [TP(*i*Pr)]BeR (*R* = Ph, *n*Bu) 6
[TP(*i*Pr)]Be(Ph) (**1a**) 6
[TP(*i*Pr)]Be(*n*Bu) (**1b**) 6
[TP(*i*Pr)O]BePh (**3d**) 7
Preparation of [TP(*i*Pr)*E*]BeCl (*E* = O, S, Se) 8
[TP(*i*Pr)*E*]BeCl (*E*= S, Se) 8
[TP(*i*Pr)S]BeCl (**3b**) 8
[TP(*i*Pr)Se]BeCl (**3c**) 9
[(Cl)Be{κ–Se(*i*Pr)$_2$PCH$_2$}μ–Se]$_2$ (5) 9
Preparation of [Ph$_2$B(μ-P(*i*Pr)$_2$CH$_2$)]$_2$ (6) 10
Preparation of [PhB{CH$_2$P(*i*Pr)$_2$}μ-P(*i*Pr)$_2$CH$_2$]$_2$ (2) 11
Preparation of [Be(*R'*)(CH$_2$P(*i*Pr)$_2$)] (*R'* = Cp, Cp*) 12
[Be(η^1-Cp)(CH$_2$P(*i*Pr)$_2$)] (**3a**) 12
[Be(η^5-Cp*)(CH$_2$P(*i*Pr)$_2$)] (**3b**) 12
Crystallographic Details 13
NMR and IR spectra 16
[TP(*i*Pr)]Be*R* (*R* = Ph, *n*Bu) 16
[TP(*i*Pr)]BePh (**1a**) 16
[TP(*i*Pr)]Be(*n*Bu) (**1b**) 19
[TP(*i*Pr)O]BePh(**3d**) 24
[TP(*i*Pr)*E*]BeCl (*E* = O, S, Se) 26
[TP(*i*Pr)O]BeCl (**3a**) 26
[TP(*i*Pr)S]BeCl (**3b**) 29
[TP(*i*Pr)Se]BeCl (**3c**) 32

[(Cl)Be{κ–Se(*i*Pr)$_2$PCH$_2$}μ–Se]$_2$ (5) 35

[Ph_2B(μ-P(*i*Pr)$_2$CH$_2$)]$_2$ (6) 38

[PhB{CH$_2$P(*i*Pr)$_2$}μ-P(*i*Pr)$_2$CH$_2$]$_2$ (2) 41

[Be(*R'*)(CH$_2$P(*i*Pr)$_2$)] (*R'* = Cp*, Cp) 43

[Be(η^5-Cp)(CH$_2$P(*i*Pr)$_2$)] (**3a**) 44

[Be(η^5-Cp*)(CH$_2$P(*i*Pr)$_2$)] (**3b**) 46

Reaction with small molecules 49

References 50

Experimental Details

Caution! Beryllium and its compounds are regarded as toxic and carcinogenic. As the biochemical mechanisms that cause beryllium associated diseases are still unknown,[1] special safety precautions are strongly advised.[2,3]

General Experimental Techniques

All manipulations were performed either under solvent vapor pressure or dry argon using a glovebox and Schlenk techniques. C_6D_6 and toluene-d_8 were dried over NaK alloy, and were subsequently vacuum distilled before storage in an argon glovebox. Toluene and *n*-pentane were dried over Na und stored over activated molecular sieve (3 Å). Lithium [tris(di-*iso*-propylphosphanylmethyl)phenylborate] ([TP(*i*Pr)]Li(thf))[4], $LiCH_2P(iPr)_2$, [TP(*i*Pr)]BeCl[5], Be(η^5-*R'*)Cl (*R'* = Cp*[6], Cp[7]), [(Et_2O)Be*R*Cl] (*R* = Ph[8], *n*Bu)[9] and red selenium[10] were prepared according to the literature. Sulfur and tellurium were purchased from commercial vendors and were sublimated prior to use. Oxygen (5.0) was used without further purification. All reactions were carried out in *J. Young* NMR tube, *Schlenk* or H-Tube which were silylated according to the procedure in reference.[2] Due to the expected extreme toxicity of the obtained compounds, no elemental analysis or mass spectrometry could be performed.

NMR spectroscopy

^{1}H, $^{9}Be\{^{1}H\}$, $^{11}B\{^{1}H\}$, $^{13}C\{^{1}H\}$, $^{31}P\{^{1}H\}$ and $^{77}Se\{^{1}H\}$ NMR spectra were recorded on Bruker Avance III HD 300 and Avance III 500 NMR spectrometers. The latter was equipped with a Prodigy Cryo-Probe. ^{1}H NMR (300 / 500 MHz) and $^{13}C\{^{1}H\}$ NMR (76 and 126 MHz) chemical shifts are given relative to the solvent signal for C_6D_6 (7.26 / 77.2 ppm) and for toluene (2.09, 7.09, 7.00, 6.98 and 20.40, 125.49, 129.24, 137.86 ppm). ^{9}Be (42 MHz) used 0.43 [M] $BeSO_4$ in D_2O as an external standard. $^{11}B\{^{1}H\}$ (96 MHz) and $^{77}Se\{^{1}H\}$ (57 MHz) NMR spectroscopy used BF_3 in diethyl ether and $SeMe_2$ as an external standard, respectively. $^{31}P\{^{1}H\}$ (122 / 202 MHz) NMR spectroscopy used 85 % H_3PO_4 as an external standard. NMR spectra were processed with the MestReNova software package.[11]

Single Crystal X-Ray Diffraction

Single crystals were selected under predried argon in perfluorinated polyether (Fomblin YR 1800, *Solvay Solexis*) and mounted using the MiTeGen MicroLoop system. X-ray diffraction data was collected using the monochromated Mo-K_α (λ = 0.71073 Å) radiation of a *Bruker* D8 Quest or of D8Venture diffractometer equipped with a microfocus source and a Photon III C14 detector. Evaluation, integration, and reduction of the diffraction data was carried out using the

APEX3[12] or APEX5[13] (Bruker D8 Quest) software suites. Multi-scan absorption correction was applied with SADABS[14]. The structures were solved with dual-space methods (SHELXT-2018/2)[15] and refined against F^2 (SHELXL-2019/1)[16] using the OLEX2[17] software packages. For the crystal data and details of the structure determination see table S1 in the supporting information. Crystal structures representations were created with the Diamond[18] software.

IR Spectroscopy

IR spectra were recorded on a *Bruker* alpha FTIR spectrometer equipped with a diamond ATR unit in an argon filled glovebox. Processing of the spectra was performed with the *OPUS*[19] software package and the MestReNova software package.[11]

Experimental Procedures

Preparation of [TP(*i*Pr)]BeR (*R* = Ph, *n*Bu)

[(Et_2O)Be*R*Cl] (1.00 eq.) was dissolved in toluene (2.00 mL) and cooled to −50 °C. To this solution, [TP(*i*Pr)]Li(thf) (1.00 eq.) in toluene (2.00 mL) was added via syringe over 20 min. The reaction mixture was stirred for 12 h and allowed to warm up to room temperature. The suspension was filtered. The filtrate was then dried under reduced pressure to obtain a colourless solid in quantitative yield (99%). [TP(*i*Pr)]BePh crystallized from a saturated toluene solution at −34 °C. [TP(*i*Pr)]Be(*n*Bu) yielded a colorless solid.

R	[(Et_2O)Be*R*Cl]	[TP(*i*Pr)]Li(thf)
Ph	62.2 mg, 0.332 mmol	185.9 mg, 0.332 mmol
*n*Bu	14.6 mg, 0.044 mmol	24.6 mg, 0.044 mmol

[TP(*i*Pr)]Be(Ph) (**1a**)

^{1}H NMR: 500 MHz, C_6D_6, δ (ppm) = 8.11 (d, $^3J_{HH}$=6.68 Hz, 1H, BeC_6H_5), 7.91 (d, $^3J_{HH}$=8.01 Hz, 2H, BC_6H_5), 7.73 (d, $^3J_{HH}$=7.82 Hz, 1H, BC_6H_5), 7.58 (t, $^3J_{HH}$=7.53 Hz, 1H, BeC_6H_5), 7.43 (t, $^3J_{HH}$=7.53 Hz, 2H, BC_6H_5), 7.34 (t, $^3J_{HH}$=7.25 Hz, 2H, C_6H_5), 7.28 (t, $^3J_{HH}$=7.53 Hz, 2H, BC_6H_5), 1.98 (hept, 6H, PCH_2), 1.22 (pseudoq, $^3J_{HH}$=7.35 Hz, 18H, CH_3), 1.06 (pseudoq, $^3J_{HH}$=7.21 Hz, 18H, CH_3), 0.95 – 0.82 (m, 6H, BCH_2P).

^{9}Be{^{1}H} NMR: 42 MHz, C_6D_6, δ (ppm) = 2.41 (s, $\omega_{1/2}$=143.3 Hz).

^{11}B{^{1}H} NMR: 96 MHz, C_6D_6, δ (ppm) = −16.1 (s).

^{13}C{^{1}H} NMR: 126 MHz, C_6D_6, δ (ppm) = 157.9 (br, s, B–C_{ipso}), 140.9 (q, $^2J_{PC}$=3.82 Hz, Be*C*), 131.4 (s, BPh–CH_o), 127.7 (s, BeC_6H$_5$), 127.4 (s, BPh–CH_m), 126.7 (s, BeC_6H$_5$), 125.2 (s, BeC_6H$_5$), 125.2 (s, BeC_6H$_5$), 124.1 (s, BPh–CH_p), 27.85 (dt, $^{1/3}J_{PC}$=7.27 Hz, P*C*H), 21.12 (s, PCH(CH_3)), 18.71 (s, PCH(CH_3)), 14.95 (dt, $^{1/3}J_{PC}$ =38.6 Hz, B*C*H$_2$P).

^{31}P{^{1}H} NMR: 122 MHz, C_6D_6, δ (ppm) = −4.48 (br, s).

IR(ATR): ν (cm^{-1}) = 3051 (br, m), 2961 (br, m), 2927 (m), 2870 (m), 1583 (w), 1458 (s), 1415 (w), 1389 (w), 1369 (m), 1246 (m), 1158 (m), 1087 (m), 1057 (m), 924 (m), 883 (m), 853 (m), 730 (w), 702 (s), 638 (w), 583 (w), 551 (w), 508 (m), 492 (s), 447 (w).

[TP(*i*Pr)]Be(*n*Bu) (**1b**)

^{1}H NMR: 500 MHz, tol-d_8, δ (ppm) = 7.78 (d, $^3J_{HH}$=7.27 Hz, 2H, C_6H_5), 7.47 (t, $^3J_{HH}$=7.38 Hz, 2H, C_6H_5), 7.24 (t, $^3J_{HH}$=7.27 Hz, 1H, C_6H_5), 1.27 (hept, $^3J_{HH}$=6.98 Hz, 18H, C*H*CH$_3$), 1.17 (t, $^3J_{HH}$=7.25 Hz, 3H, CH_2CH_2CH$_3$),

1.13–1.05 (m, 18H, CHCH_3), 0.78 (s, 6H, BCH_2), 0.44–0.32 (m, 2H, BeCH_2CH$_2$).

^{9}Be{^{1}H} NMR: 42 MHz, tol-d_8, δ (ppm) = 2.50 (s, $\omega_{1/2}$=96.6 Hz). C_6D_6, δ (ppm) = 2.36 (s, $\omega_{1/2}$=22.2 Hz).

^{11}B{^{1}H} NMR: 96 MHz, tol-d_8, δ (ppm) = −16.1(s).

^{13}C{^{1}H} NMR: 126 MHz, tol-d_8, δ (ppm) = 131.3 (s, BPh-CH_o), 127.4 (s, BPh-CH_m), 123.9 (s, BPh-CH_p), 35.7 (q, $^2J_{BeC}$=5.43 Hz, BeCH$_2$CH$_2$), 31.49 (s, CH$_2$CH$_2$CH$_3$). 27.81 (dt, $^{1/3}J_{PC}$=5.85 Hz, PCH), 20.91 (q, J_{BeC}=7.54 Hz, BeCH$_2$), 18.44 (s, PCH(CH$_3$)), 14.98 (dt, $^{1/3}J_{PC}$=37.6 Hz, BCH$_2$P), 14.34 (s, CH$_2$CH$_3$).

^{31}P{^{1}H} NMR: 122 MHz, tol-d_8, δ (ppm) = −2.70 (br, s). C_6D_6, δ (ppm) = −2.81 (s).

IR(ATR): ν (cm^{-1}) = 2949 (m), 2896 (s), 2870 (s), 1595 (s), 1460 (w), 1428 (w), 1389 (w), 1371 (w), 1260 (w), 1057 (m), 1028 (m), 916 (w), 881 (s), 853 (w), 736 (w), 702 (w), 626 (w), 583 (s), 551 (w), 510 (w), 465 (w), 451 (w), 436 (w), 426 (w).

[TP(*i*Pr)O]BePh (**3d**)

In a *J. Young* NMR tube [TP(*i*Pr)]BePh (14.2 mg, 0.025 mmol) was dissolved in 500 µL benzene. The argon atmosphere was removed and then 2 bar of oxygen was added. The reaction mixture was shaken at ambient temperature. The mixture was then dried under reduced pressure to obtain a colorless solid in quantitative yield.

^{1}H NMR: 300 MHz, C_6D_6, δ (ppm) = 7.69 (d, $^3J_{HH}$=7.63 Hz, 3H, C_6H_5), 7.51 (t, $^3J_{HH}$=7.63 Hz, 2H, C_6H_5), 7.50–7.43 (m, 3H, C_6H_5), 7.45–7.38 (m, 3H, C_6H_5), 7.36–7.30 (m, 3H, C_6H_5), 2.12 (hept, $^3J_{HH}$=7.44, 6H, PCH), 1.06 (dd, $^3J_{PH}$=14.50, $^3J_{HH}$=7.25, 18H, CH_3), 1.06 (dd, $^3J_{PH}$=14.50 Hz, $^3J_{HH}$=7.25 Hz, 18H; CH_3), 0.83–0.69 (m, 6H, CH_2).

^{9}Be{^{1}H} NMR: 42 MHz, C_6D_6, δ (ppm) = 0.89 (s).

^{11}B{^{1}H} NMR: 96 MHz, C_6D_6, δ (ppm) = −15.4 (s).

^{31}P{^{1}H} NMR: 121 MHz, C_6D_6, δ (ppm) = 73.5 (s).

IR(ATR): ν (cm^{-1}) = 2959 (w), 2919 (m), 2874 (w), 2849 (w), 1593 (m), 1493 (w), 1462 (m), 1428 (w), 1387 (w), 1295 (w), 1271 (w), 1246 (w), 1226 (w), 1087 (s), 1026 (m), 993 (w), 928 (w), 883 (s), 753 (s), 702 (w), 675 (w), 600 (w), 500 (w), 471 (w), 453 (w), 434 (w), 420 (w), 404 (w).

Preparation of [TP(*i*Pr)*E*]BeCl (*E* = O, S, Se)

[TP(*i*Pr)O]BeCl (**3a**)

In a *J. Young* NMR tube [TP(*i*Pr)]BeCl (30.1 mg, 0.053 mmol) was dissolved in 500 µL benzene. The argon atmosphere was removed and then 2 bar of oxygen was added. The reaction mixture was shaken at ambient temperature. The mixture was then dried under reduced pressure to obtain a colorless solid in quantitative yield.

**^{1}H NMR:**	300 MHz, C_6D_6, δ (ppm) = 7.72–7.61 (m, 2H, C_6H_5), 7.56–7.46 (m, 2H, C_6H_5), 7.42 (s, 1H, C_6H_5), 2.14 (hep, $^3J_{HH}$=6.95 Hz, 6H, PC*H*), 1.14 (dd, $^3J_{PH}$=14.49 Hz, $^3J_{HH}$ = 7.31 Hz, 18H, C*H*$_3$), 0.92 (dd, $^3J_{PH}$=15.26 Hz, $^3J_{HH}$=7.36 Hz, 18H, C*H*$_3$), 0.84–0.72 (m, 6H, C*H*$_2$).
**^{9}Be{^{1}H} NMR:**	42 MHz, C_6D_6, δ (ppm) = 1.79 (s).
**^{11}B{^{1}H} NMR:**	96 MHz, C_6D_6, δ (ppm) = −15.1 (s).
**^{13}C{^{1}H} NMR:**	126 MHz, C_6D_6, δ (ppm) = 130.3 (s, BPh-C_o), 127.1 (s, BPh-C_m), 124.2 (s, BPh-C_p), 28.1 (s, P*C*H), 27.6 (s, PCH(*C*H$_3$)), 16.5 (s, PCH(*C*H$_3$)), 15.7–14.8 (br, m, B*C*H$_2$P).
**^{31}P{^{1}H} NMR:**	121 MHz, C_6D_6, δ (ppm) = 75.0 (s).
**IR(ATR)**:	ν (cm^{-1}) = 2964 (w), 2935 (w), 2874 (w), 1626 (w), 1603 (m), 1464 (m), 1428 (w), 1413 (w), 1389 (w), 1334 (w), 1299 (w), 1273 (w), 1165 (s), 1116 (s), 1079 (w), 1028 (w), 975 (m), 887 (m), 779 (m), 749 (w), 726 (s), 702 (s), 677 (w), 618 (w), 598 (m), 575 (w), 534 (w), 500 (w), 481 (w), 453 (w), 443 (w), 434 (w), 412 (w).

[TP(*i*Pr)*E*]BeCl (*E*= S, Se)

Benzene was added to [TP(*i*Pr)]BeCl und E_n giving a colorless suspension. Following sonication or stirring at ambient temperature for 8 h, the suspension was filtered. The filtrate was then dried under reduced pressure to obtain a colorless solid in quantitative yield.

E_n	[TP(*i*Pr)]BeCl	E_n	benzene
S_8	30.0 mg, 0.057 mmol	3.80 mg, 0.11 mmol	3.00 mL
Se_8	30.0 mg, 0.057 mmol	9.50 mg, 0.12 mmol	3.00 mL

[TP(*i*Pr)S]BeCl (**3b**)

**^{1}H NMR:**	300 MHz, C_6D_6, δ (ppm) = 7.95 (d, $^3J_{HH}$=6.81, 1H, C_6H_6), 7.74–7.68 (m, 2H, C_6H_6), 7.03 (t, $^3J_{HH}$=6.68 Hz, 2H, C_6H_6), 1.79–1.59 (m, 6H, C*H*), 0.99 (dd, $^3J_{HH}$=6.95 Hz, $^3J_{PH}$=2.04 Hz, 18H, C*H*$_3$), 0.93 (dd, $^3J_{HH}$=6.99, $^3J_{PH}$=1.91 Hz, 18H, C*H*$_3$), 0.72 (td, $^2J_{PH}$=17.7 Hz, $^3J_{HH}$=7.04, 6H, C*H*$_2$).

^{9}Be{^{1}H} NMR: 42 MHz, C_6D_6, δ (in ppm) = 6.08 (s).

^{11}B{^{1}H} NMR: 96 MHz, C_6D_6, δ (in ppm) = −13.3 (s).

^{13}C{^{1}H} NMR: 126 MHz, C_6D_6, δ (ppm) = 146.1 (s, C*H*), 133.8 (s, CH_o), 129.3 (s, CH_m), 125.7 (s, CH_m), 28.3 (s, PCH(CH_3)), 28.3 (s, PCH(CH_3)), 21.5 (s, P*C*H), 16.5 (s, PCH(CH_3)), 15.9 (s, BCH_2P).

^{31}P{^{1}H} NMR: 121 MHz, C_6D_6, δ (ppm) = 64.1 (s).

IR(ATR): ν (cm^{-1}) = 2961 (m), 2931 (w), 2872 (w), 1601 (w), 1460 (m), 1364 (s), 1344 (s), 1307 (w), 1250 (w), 1179 (w), 1087 (w), 1026 (w), 930 (m), 881 (s), 810 (w), 779 (w), 673 (s), 596 (w), 579 (w), 547 (w), 498 (w), 463 (w), 443 (w), 416 (w).

[TP(*i*Pr)Se]BeCl (**3c**)

^{1}H NMR: 300 MHz, C_6D_6, δ (ppm) = 7.71 (d, $^{3}J_{HH}$=7.81 Hz, C_6H_6), 7.50 (t, $^{3}J_{HH}$=7.63, 1H C_6H_6), 7.07–6.96 (m, 2H C_6H_6), 3.06 (d, $^{3}J_{PH}$=16.3 Hz, 6H, CH_2), 1.87–1.57 (m, 6H, C*H*), 0.98 (d, $^{3}J_{HH}$=6.90 Hz, 18H, CH_3), 0.92 (d, $^{3}J_{HH}$=6.99 Hz, 18H, CH_3).

^{9}Be{^{1}H} NMR: 42 MHz, C_6D_6, δ (ppm) = 4.83 (m).

^{11}B{^{1}H} NMR: 96 MHz, C_6D_6, δ (ppm) = −15.4 (s).

^{13}C{^{1}H} NMR: 126 MHz, C_6D_6, δ (ppm) = 145.6 (s, C*H*), 134.1(s, CH_o), 129.3 (s, CH_m), 125.7(s, CH_m), 29.3 (s, PCH(CH_3)), 29.0(s, PCH(CH_3)), 16.9 (s, PCH(CH_3)), 16.4 (s, BCH_2P).

^{31}P{^{1}H} NMR: 121 MHz, C_6D_6, δ (ppm) = 50.33 (s, $^{1}J_{PSe}$=651 Hz).

^{77}Se{^{1}H} NMR: 57 MHz, C_6D_6, δ (ppm) = −357.7 (d, $^{1}J_{PSe}$=651 Hz).

IR(ATR): ν (cm^{-1}) = 3035 (w), 2961 (s), 2929 (m), 2870 (m), 1593 (w), 1460 (s), 1430 (w), 1381 (m), 1364 (m), 1244 (m), 1154 (m), 1079 (m), 1032 (s), 928 (m), 881 (m), 730 (s), 706 (m), 679 (m), 663 (m), 653 (m), 636 (m), 553 (w), 485 (m), 424 (w).

[(Cl)Be{κ–Se(*i*Pr)$_2$PCH$_2$}μ–Se]$_2$ (5)

Benzene was added to [TP(*i*Pr)]BeCl (13.4 mg, mmol) und Se_8 (6.3 mg, mmol) giving a colorless suspension. Following sonication and stirring at ambient temperature for 24 h, the suspension was filtered. The filtrate was then dried under reduced pressure to obtain a colorless solid. The products crystallized from a saturated toluene solution at −34 °C.

^{1}H NMR: 300 MHz, C_6D_6, δ (ppm) = 3.06 (d, $^{2}J_{PH}$=16.26 Hz, 4H, CH_2P), 1.71 (hept, $^{3}J_{HH}$=6.99 Hz, 6H, C*H*CH_3), 0.98 (d, $^{3}J_{HH}$=6.90 Hz, 12H, CHCH_3), 0.92 (d, $^{3}J_{HH}$=6.99 Hz, 12H, CHCH_3).

$^{9}Be\{^{1}H\}$ NMR: 42 MHz, C_6D_6, δ (ppm) = 7.40 (br, s, $\omega_{1/2}$=33.6 Hz)

$^{31}P\{^{1}H\}$ NMR: 121 MHz, C_6D_6, δ (ppm) = 60.26 (s, $^{1}J_{PSe}$=652 Hz)

$^{77}Se\{^{1}H\}$ NMR: 57 MHz, C_6D_6, δ (ppm) = −357.3 (d, $^{1}J_{PSe}$=652 Hz).

IR(ATR): ν (cm^{-1}) = 2961 (w), 2927 (w), 2868 (w), 1589 (w), 1456 (w), 1434 (m), 1381 (m), 1364 (m), 1240 (s), 1191 (w), 1150 (s), 1120 (w), 1081 (w), 1065 (w), 1028 (s), 997 (w), 979 (w), 922 (s), 879 (s), 822 (m), 781 (m), 757 (w), 728 (s), 704 (w), 691 (w), 657 (w), 645 (s), 618 (w), 577 (w), 498 (w), 483 (w), 469 (w), 457 (m), 449 (m), 424 (s).

Preparation of $[Ph_2B(\mu\text{-}P(\mathit{i}Pr)_2CH_2)]_2$ (6)

Benzene was added to [TP(*i*Pr)]BeCl (30.2 mg, 0.057 mmol) und Te (7.28 mg, 0.075 mmol) giving a colorless suspension. Following sonication and reflux at 100 °C temperature for 24 h, the suspension was filtered. The filtrate was then dried under reduced pressure to obtain a colorless solid. **5** crystallized from a saturated toluene solution at −34 °C.

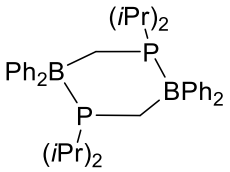

^{1}H NMR: 300 MHz, C_6D_6, δ (ppm) = 7.73 (d, $^{3}J_{HH}$=8.08 Hz, 8H, C_6H_5), 7.28 (t, $^{3}J_{HH}$=7.31 Hz, 8H, C_6H_5), 7.19 (d, $^{3}J_{HH}$=7.18 Hz, 4H, C_6H_5), 2.21–2.03 (m, 6H, $CHCH_3$), 1.92–1.76 (m, 4H, CH_2), 0.68–0.53 (m, 24H, $CHCH_3$).

$^{11}B\{^{1}H\}$ NMR: 96 MHz, C_6D_6, δ (ppm) = −10.6 (br, s).

$^{13}C\{^{1}H\}$ NMR: 126 MHz, C_6D_6, δ (ppm) = 135.7 (s, C_6H_6), 135.6 (s, C_6H_6), 129.3 (s, C_6H_6), 127.4 (s, C_6H_6), 125.7 (s, C_6H_6), 25.37 (s, CH_3), 25.03 (s, CH_3), 18.79 (m, CH_2), 18.54 (d, $^{1}J_{PC}$=4.69 Hz, $CHCH_3$).

$^{31}P\{^{1}H\}$ NMR: 121 MHz, C_6D_6, δ (ppm) = 7.46 (br, s).

IR(ATR): ν (cm^{-1}) = 3063 (w), 3043 (w), 2964 (w), 2931 (w), 2872 (w), 1595 (w), 1485 (w), 1462 (w), 1428 (w), 1393 (w), 1371 (w), 1232 (s), 1179 (m), 1124 (s), 1067 (w), 1028 (w), 981 (w), 928 (m), 885 (m), 806 (w), 753 (w), 726 (w), 700 (s), 661 (w), 618 (w), 534 (w), 512 (w), 500 (w), 475 (w), 453 (w), 428 (w), 420 (w), 412 (w).

Preparation of [PhB{CH$_2$P(*i*Pr)$_2$}μ-P(*i*Pr)$_2$CH$_2$]$_2$ (2)

Benzene was added to [TP(*i*Pr)]Li(thf) (108.9 mg, 0.185 mmol) und Be(η^5-cp*)Cl (33.2 mg, 0.185mmol) giving a colorless suspension. Following sonication and stirring at ambient temperature for 24 h, the suspension was filtered. The filtrate was then dried under reduced pressure to obtain a colorless solid. **4** crystallized from a saturated toluene solution at −34 °C.

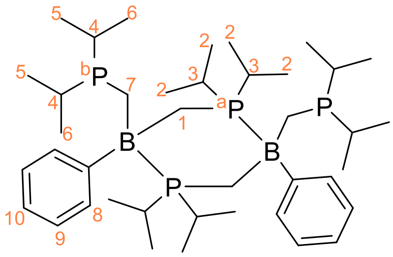

^{1}H NMR: 300 MHz, C_6D_6, δ (ppm) = 7.77 (dd, $^3J_{HH}$=6.54 Hz, $^4J_{HH}$=1.63 Hz, 4H, $^{8/9}$C*H*), 7.34 (t, $^3J_{HH}$=7.54 Hz, 4H, $^{8/9}$C*H*), 7.21 (dd, $^3J_{HH}$=7.18 Hz, $^4J_{HH}$=1.64 Hz, 2H, ^{10}C*H*), 2.82 (hept, $^3J_{HH}$=7.58 Hz, 3H, $^{3/4}$C*H*), 2.14 (d, $^3J_{HH}$=4.36, 1H, $^{3/4}$C*H*), 1.98–1.84 (m, 6H, $^{3/4}$C*H*), 1.65 (dd, $^3J_{PH}$=14.4 Hz, $^3J_{HH}$=7.31 Hz, 6H, $^{5/6}$C*H*$_3$), 1.51 (dd, $^3J_{PH}$=10.8 Hz, $^3J_{HH}$=7.13 Hz, 6H, $^{5/6}$C*H*$_3$), 1.44–1.35 (m, 4H, $^{1/7}$CH$_2$), 1.30–1.09 (m, 12H, ^{2}C*H*$_3$), 1.09–1.06 (m, 4H, $^{1/7}$CH$_2$), 1.03–0.96 (m, 12H, ^{2}C*H*$_3$), 0.78 (dd, $^3J_{PH}$=11.2 Hz, $^3J_{HH}$=6.99 Hz, 6H, $^{5/6}$C*H*$_3$), 0.56 (dd, $^3J_{PH}$=13.4 Hz, $^3J_{HH}$=7.08 Hz, 6H; $^{5/6}$C*H*$_3$).

^{11}B{^{1}H} NMR: 96 MHz, C_6D_6, δ (ppm) = −12.8 (s).

^{13}C{^{1}H} NMR: 126 MHz, C_6D_6, δ (ppm) = 134.3 (s, $^{8/9}$*C*H), 134.2 (s, $^{8/9}$*C*H), 127.3 (s, ^{10}*C*H), 125.6 (s, $^{8/9}$*C*H), 125.6 (s, $^{8/9}$*C*H), 25.34 (d, $^2J_{PC}$=17.0 Hz, $^{3/4}$P*C*H), 24.81 (d, $^2J_{PC}$=18.8 Hz, $^{3/4}$P*C*H), 24.39 (d, $^2J_{PC}$=23.2 Hz, $^{3/4}$P*C*H), 23.23 (d, $^2J_{PC}$=17.6 Hz, $^{3/4}$P*C*H), 22.21 (d, $^2J_{PC}$=21.4 Hz, $^{3/4}$P*C*H), 21.54 (d, $^2J_{PC}$=16.4 Hz, $^{3/4}$P*C*H), 21.20 (d, $^2J_{PC}$=12.3 Hz, P*C*H), 20.18 (s, $^{5/6}$*C*H$_3$), 20.14 (s, $^{5/6}$*C*H$_3$), 18.09 (s, ^{2}*C*H$_3$), 18.07–17.95 (m, $^{1/7}$*C*H$_2$).

^{31}P{^{1}H} NMR: 121 MHz, C_6D_6, δ (ppm) = 9.31 (br, s, ^{a}*P*CH$_2$), 3.17 (d, $^3J_{PP}$=33.3 Hz, ^{b}*P*CH$_2$).

IR(ATR): ν (cm^{-1}) = 3070 (br, w), 2998 (w), 2964 (m), 2923 (m), 2886 (m), 2864 (m), 1589 (w), 1485 (w), 1458 (m), 1428 (w), 1383 (w), 1358 (w), 1303 (w), 1228 (s), 1173 (w), 1122 (s), 1034 (w), 981 (s), 926 (w), 875 (m), 797 (m), 773 (m), 742 (m), 698 (m), 647 (m), 583 (m), 532 (s), 483 (m), 432 (w).

Preparation of [Be(*R'*)(CH_2P(*i*Pr)$_2$)] (*R'* = Cp, Cp*)

Benzene was added to Be(η^5-*R'*)Cl (1.00 eq) und LiCH_2P(*i*Pr)$_2$ (1.00 eq) giving a colorless suspension. Following stirring at ambient temperature for 2 h, the suspension was filtered. The filtrate was then dried under reduced pressure to obtain a colorless solid in quantitative yield.

R'	Be(η^5-*R*)Cl	LiCH_2P(*i*Pr)$_2$
Cp*	20.2 mg, 0.112 mmol	14.9 mg, 0.108 mmol
Cp	12.3 mg, 0.112 mmol	15.0 mg, 0.109 mmol

[Be(η^1-Cp)(CH_2P(*i*Pr)$_2$)] (**3a**)

^{1}H NMR: 300 MHz, C_6D_6, δ (ppm) = 5.71 (s, 5H, CH^{Cp}), 1.53 (heptd, $^3J_{HH}$=6.95 Hz, 1.73, 2H, PC*H*), 1.11 (ddd, $^3J_{HH}$=11.76 Hz, $^3J_{HH}$=7.08 Hz, $^3J_{PH}$=2.75 Hz, 12H, CH_3), −0.77 (d, $^2J_{PH}$ =1.91 Hz, 2H, CH_2).

^{9}Be{^{1}H} NMR: 42 MHz, C_6D_6, δ (ppm) = −19.6 (s, $\omega_{1/2}$=21.9 Hz).

^{13}C{^{1}H} NMR: 126 MHz, C_6D_6, δ (ppm) = 104.01 (s, C^{cp*}), 26.15 (d, $^1J_{PC}$=16.73 Hz, P*C*H), 20.60 (d, $^2J_{PC}$=16.43 Hz, *C*H_3), 19.24 (d, $^2J_{PC}$=9.98 Hz, *C*H_3), −15.11 (br, s, Be*C*H_2P).

^{31}P{^{1}H} NMR: 121 MHz, C_6D_6, δ (ppm) = 9.04 (s).

IR(ATR): ν (cm^{-1}) = 2947 (w), 2925 (w), 2890 (w), 2864 (w), 1460 (w), 1362 (w), 1128 (w), 1057 (m), 1006 (s), 959 (m), 881 (w), 800 (w), 749 (s), 659 (w), 512 (w), 424 (w), 412 (w).

[Be(η^5-Cp*)(CH_2P(*i*Pr)$_2$)] (**3b**)

^{1}H NMR: 300 MHz, C_6D_6, δ (ppm) = 1.79 (s, 15H, CH_3^{cp*}), 1.46 (heptd, $^3J_{HH}$=7.04 Hz, $^2J_{PH}$=2.13 Hz, 2H, PC*H*), 1.13 (ddd, $^3J_{HH}$=11.17, $^3J_{HH}$=6.99 Hz, $^3J_{PH}$=2.91 Hz, 12H, CH_3), −0.72 (d, $^2J_{PH}$=1.45 Hz, 2H, BeCH_2P).

^{9}Be{^{1}H} NMR: 42 MHz, C_6D_6, δ (in ppm) = −17.0 (s, $\omega_{1/2}$=23.3 Hz).

^{13}C{^{1}H} NMR: 126 MHz, C_6D_6, δ (ppm) = 108.75 (s, C^{cp*}), 26.52 (d, $^1J_{PC}$=17.02 Hz, P*C*H), 20.04 (d, $^2J_{PC}$=14.67 Hz, *C*H_3), 19.62 (d, $^2J_{PC}$=12.32 Hz, *C*H_3), 9.26 (s, CH_3^{cp*}), −12.89 (br, s, Be*C*H_2P).

^{31}P{^{1}H} NMR: 121 MHz, C_6D_6, δ (ppm) = 5.30 (s).

IR(ATR): ν (cm^{-1}) = 2943 (s), 2919 (s), 2862 (s), 1456 (m), 1381 (m), 1358 (w), 1246 (w), 1065 (s), 1008 (s), 983 (s), 961 (s), 922 (s), 879 (s), 795 (w), 773 (w), 742 (w), 724 (w), 700 (w), 661 (w), 606 (w), 581 (w), 540 (w), 524 (w), 471 (w), 457 (w), 439 (w), 420 (w), 412 (w).

Crystallographic Details

Table S1: Crystal data and details of the structure determination for ***1a****,* ***2, 5*** *and* ***6****.*

Compound	[TP(iPr)]BePh	[(Cl)Be{κ–Se(iPr)$_2$PCH$_2$}μ–Se]$_2$	[PhB{CH$_2$P(iPr)$_2$}μ-P(iPr)$_2$CH$_2$]$_2$	[Ph$_2$B(μ-P(iPr)$_2$CH$_2$)]$_2$
Empirical formula	$C_{33}H_{58}BBeP_3$	$C_{14}H_{32}Be_2P_2Cl_2Se_4$	$C_{40}H_{74}B_2P_4$	$C_{52}H_{68}B_2P_2$
Relative molecular mass	567.52	667.09	700.49	776.62
Crystal system	orthorhombic	monoclinic	monoclinic	monoclinic
Space group (number)	$P2_12_12_1$ (19)	$P2_1/n$ (14)	$C2_1/c$ (15)	$P2_1/c$ (14)
Radiation	MoK_α (λ=0.71073 Å)	MoK_α (λ=0.71073 Å)	MoK_α (λ=0.71073 Å)	MoK_α (λ=0.71073 Å)
T [K]	100	100	100	100
a [Å]	9.8528(4)	7.9964(7)	38.1478(13)	11.2401(5)
b [Å]	17.8892(7)	14.4516(12)	10.2272(3)	9.9582(4)
c [Å]	18.8450(8)	10.6168(10)	22.9994(7)	20.2805(9)
α [°]	90	90	90	90
β [°]	90	93.650(3)	108.1270(10)	101.8270(10)
γ [°]	90	90	90	90
V [Å^3]	3321.6(2)	1224.40(19)	8527.8(5)	2221.83(17)
Z	4	2	8	2
F(000) [e$^-$]	1240	648.0	3072	840.0
ρ_{calc} [gcm^{-3}]	1.135	6.339	1.091	1.161
μ [mm^{-1}]	0.199	0.203	0.203	0.133
2θ range [°]	4.32 to 52.89	4.768 to 53.69	4.14 to 52.74	4.104 to 50.05
Index ranges	−12 ≤ h ≤ 12<br>−22 ≤ k ≤ 22<br>−23 ≤ l ≤ 23	−10 ≤ h ≤ 10<br>−18 ≤ k ≤ 18<br>−13 ≤ l ≤ 13	−47 ≤ h ≤ 47<br>−12 ≤ k ≤ 12<br>−28 ≤ l ≤ 28	−13 ≤ h ≤ 13<br>−11 ≤ k ≤ 11<br>−24 ≤ l ≤ 24
Reflections collected / unique	93943 / 6840	59719/ 2618	218714 / 8724	44687 / 3917
Restraints / Parameters	6 / 407	0 / 113	0 / 431	0 / 258
R_{int}	0.0957	0.0555	0.0577	0.0774
R_1 [I≥2σ(I)]	0.0694	0.0257	0.0494	0.0958
R_1 [all data]	0.0704	0.0330	0.0532	0.1079
wR_2 [I≥2σ(I)]	0.1509	0.0564	0.1121	0.1788
wR_2 [all data]	0.1514	0.0587	0.1141	0.1849
S	1.174	1.166	1.156	1.298
Δρ$_{min}$, Δρ$_{max}$ [e·Å^{-3}]	−0.42/0.42	−0.38 / 0.58	−0.37 / 0.77	−0.67 / 0.79
Flack parameter	*0.1(2)*			

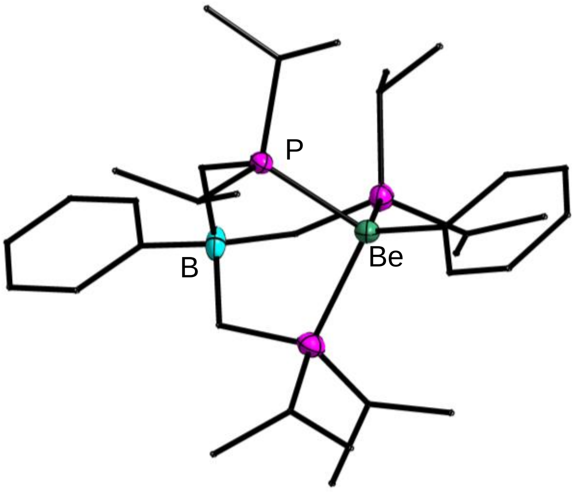

Figure S1. Molecular structure of [TP(iPr)]BePh in the solid state. Ellipsoids shown at 70 % probability at 100 K. Hydrogen atoms are omitted for clarity. Carbon atoms are shown as wire frames for clarity. Selected bond lengths (Å) and angles (°): Be—C1 1.7655(2), Be—P1 2.2642(4), Be—P2 2.2292(6), Be—P3 2.2252(1); P3—Be—C1 119.7(4), P1—Be—C1 121.6(4), P2—Be—C1 119.4(4), B—Be—C1 178.4(4).

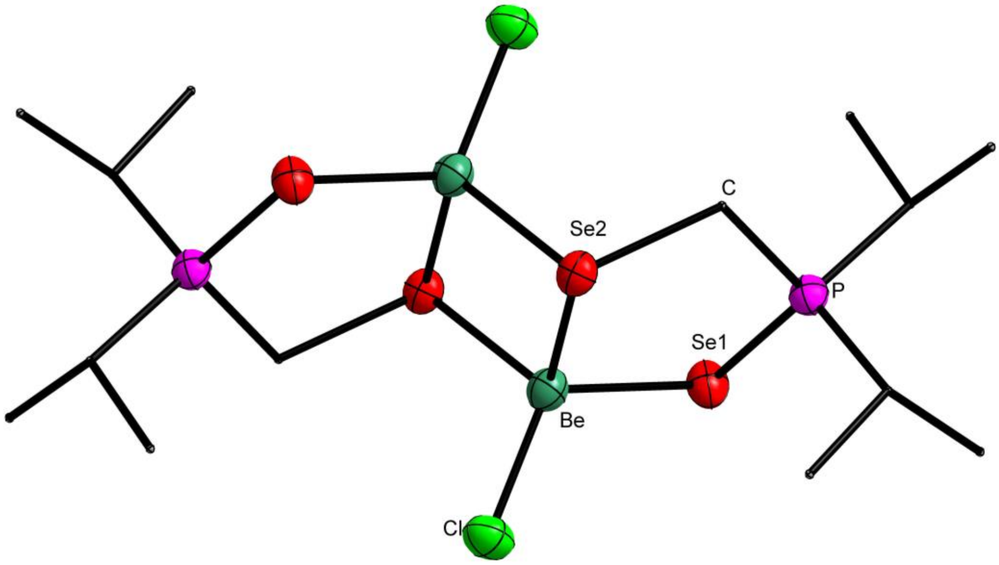

Figure S2. Molecular structure of [(Cl)Be{κ–Se(iPr)$_2$PCH$_2$}μ–Se]$_2$ in the solid state. Ellipsoids shown at 70 % probability at 100 K. Hydrogen atoms are omitted for clarity. Carbon atoms are shown as wire frames for clarity. Selected bond lengths (Å) and angles (°): Be—Se1 2.257(4), Be—Se2 2.274(4), 2.2909(1), Se1—P 2.1529(8), Be—Cl 1.946(4), P—C1 1.809(3); Se1—Be—Se2 106.1(2), Se1—Be—Cl 117.4(2), Se2—Be—Cl 111.7(2), Be—Se2—Be 76.1(2).

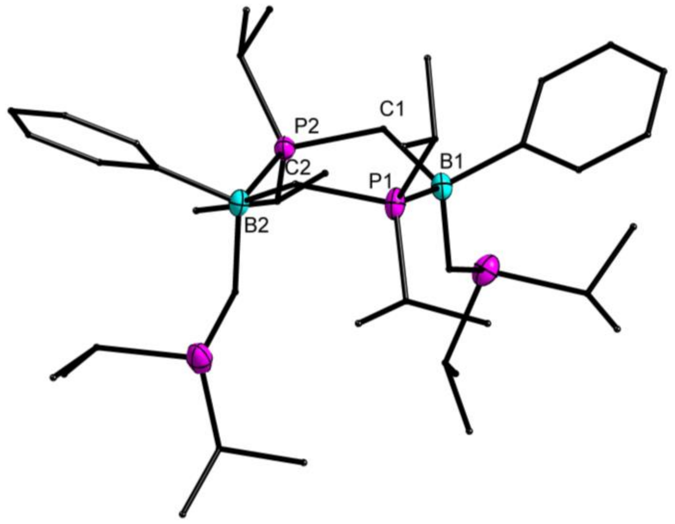

Figure S3. Molecular structure of [PhB{CH$_2$P(iPr)$_2$}μ-P(iPr)$_2$CH$_2$]$_2$ in the solid state. Ellipsoids shown at 70 % probability at 100 K. Hydrogen atoms are omitted for clarity. Carbon atoms are shown as wire frames for clarity. Selected bond lengths (Å) and angles (°): P1—B1 2.0356(1), P1—C2 1.8149(7), P1—C2 1.8191(8), P2—B2 2.0408(2), P2—C1 1.8149(7), P2—C2 1.8191(8), B1—C5 1.6418(3), B2—C11 1.6434(5); C1—B1—P1 105.03(2), C1—B1—C5 108.51(5), C2—B2—P2 108.86(2), C2—B2—C11 109.47(2).

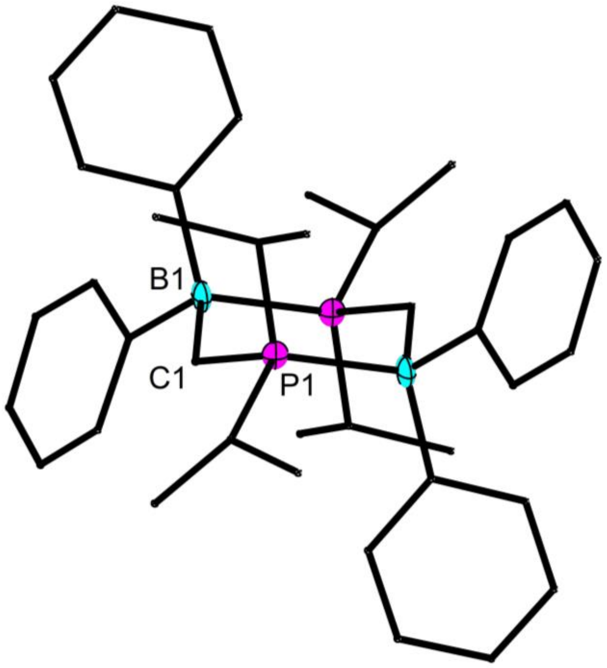

Figure S4. Molecular structure of [Ph$_2$B(μ-P(iPr)$_2$CH$_2$)]$_2$ in the solid state. Ellipsoids shown at 70 % probability at 100 K. Hydrogen atoms are omitted for clarity. Carbon atoms are shown as wire frames for clarity. Selected bond lengths (Å) and angles (°): P1—B1 2.0188(0), P1—C2 1.8640(7), P1—C2 1.8184(8), B1—C1 1.6635(4), B1—C8 1.6261(4), B1—C14 1.6403(4); B1— C1—P1 124.9(3), P1—B1—C1 105.1(3), C5—P1—C2 103.3(2), C8—B2—C14 110.7(3).

NMR and IR spectra

[TP(*i*Pr)]Be*R* (*R* = Ph, *n*Bu)

[TP(*i*Pr)]BePh (**1a**)

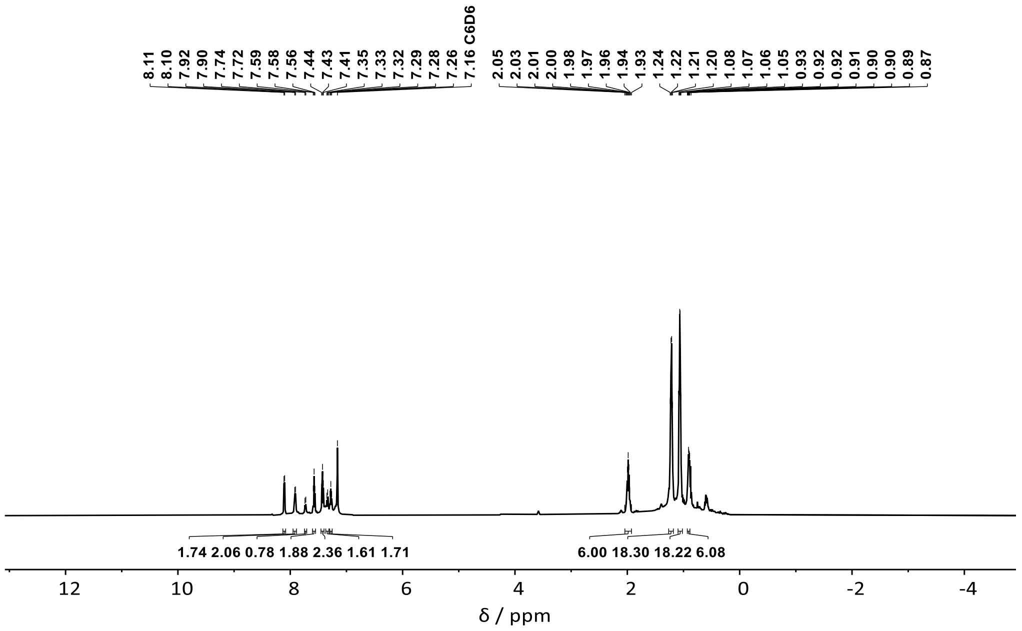

Figure S5. ^{1}H NMR spectrum of [TP(iPr)]BePh in C_6D_6.

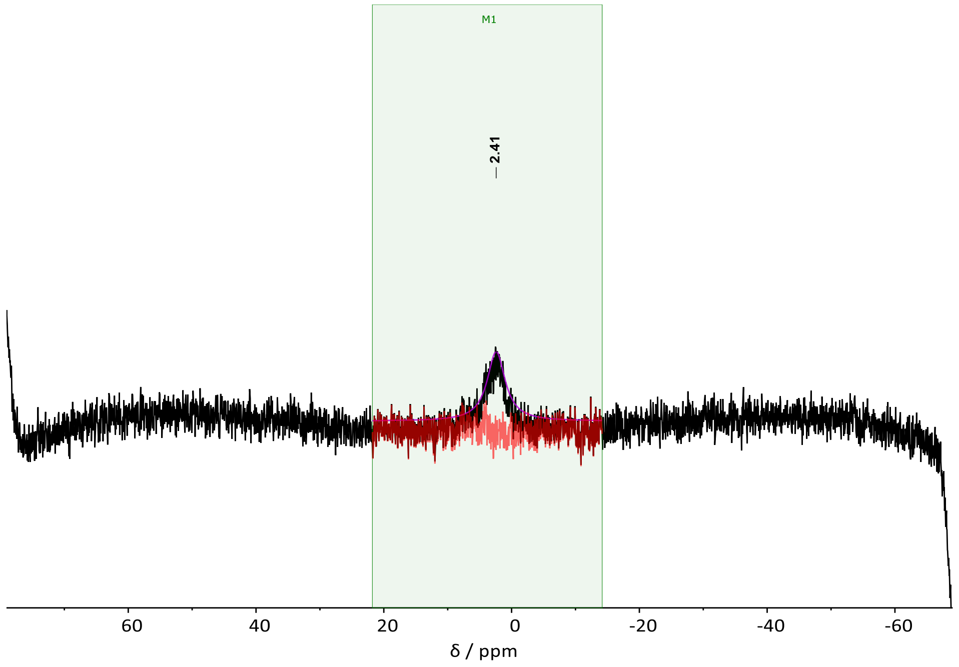

Figure S6. ^{9}Be{^{1}H} NMR spectrum of [TP(iPr)]BePh in C_6D_6.

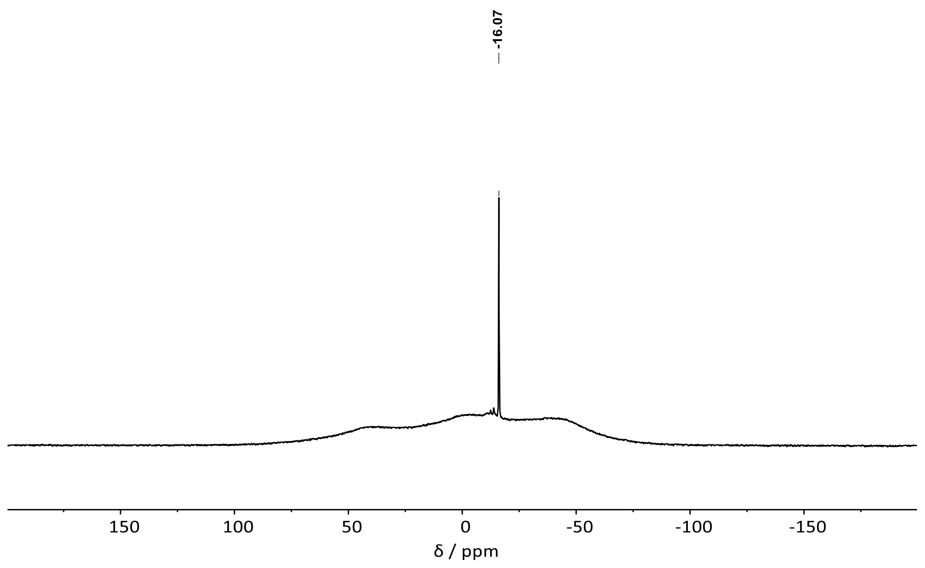

Figure S7. $^{11}B\{^1H\}$ NMR spectrum of [TP(iPr)]BePh in C_6D_6.

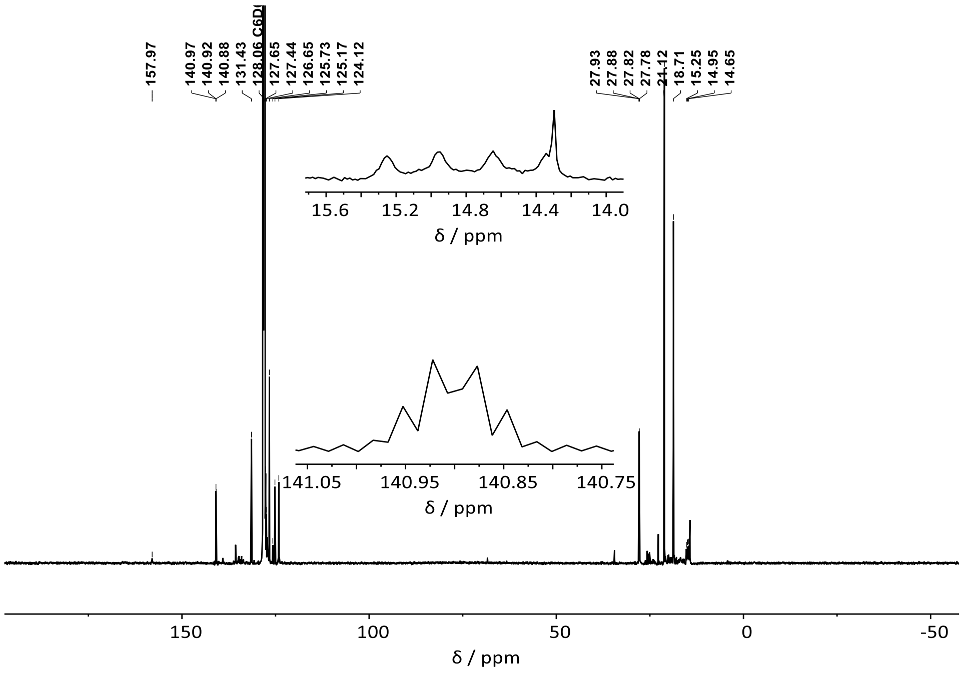

Figure S8. $^{13}C\{^1H\}$ NMR spectrum of [TP(iPr)]BePh in C_6D_6.

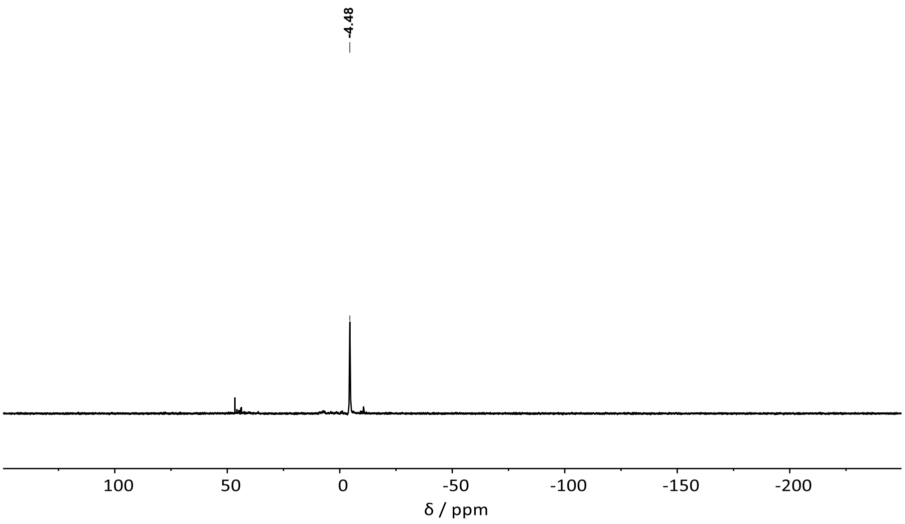

Figure S9. $^{31}P\{^{1}H\}$ *NMR spectrum of [TP(iPr)]BePh in* C_6D_6.

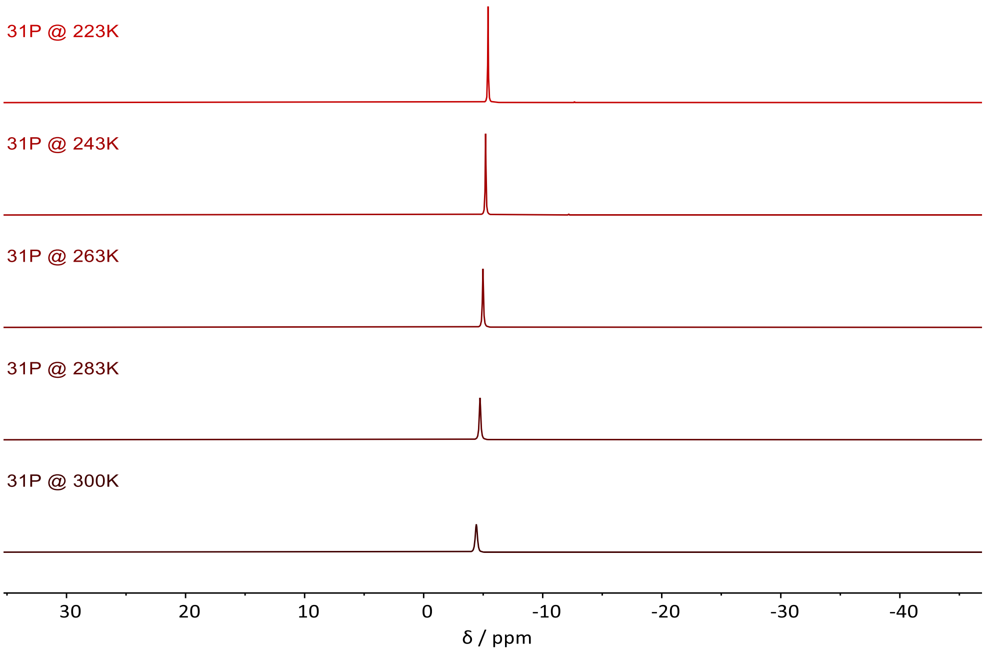

Figure S10. $^{31}P\{^{1}H\}$ *NMR spectra temperature experiment of [TP(iPr)]BePh in toluene-*d_8.

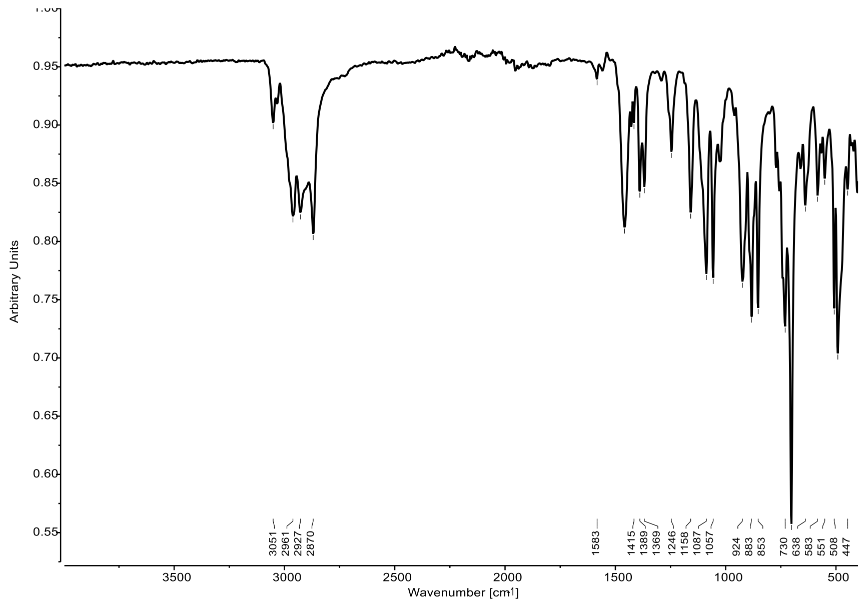

Figure S11. IR spectrum of [TP(iPr)]BePh.

[TP(*i*Pr)]Be(*n*Bu) (**1b**)

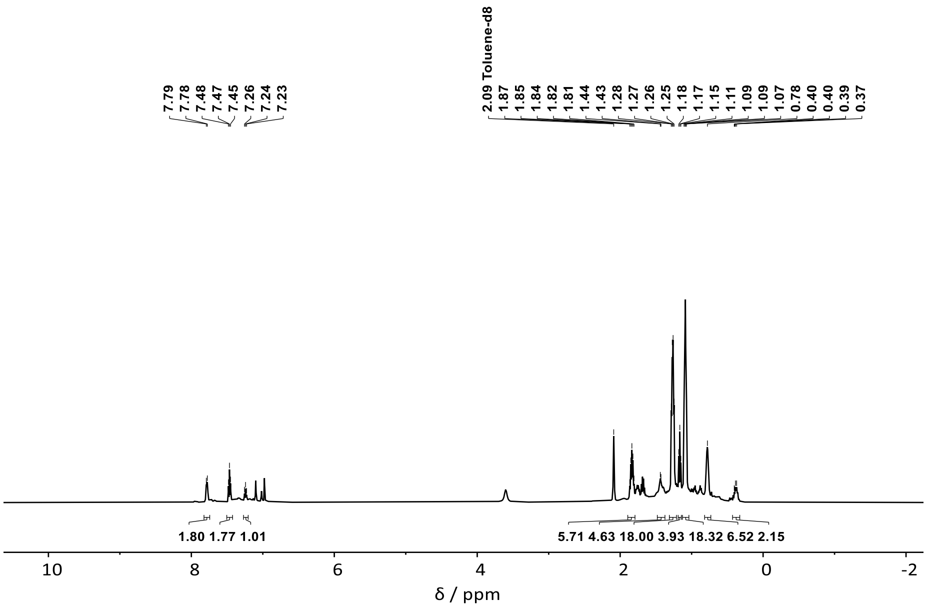

Figure S12. ^{1}H NMR spectrum of [TP(iPr)]Be(nBu) in toluene-d_8.

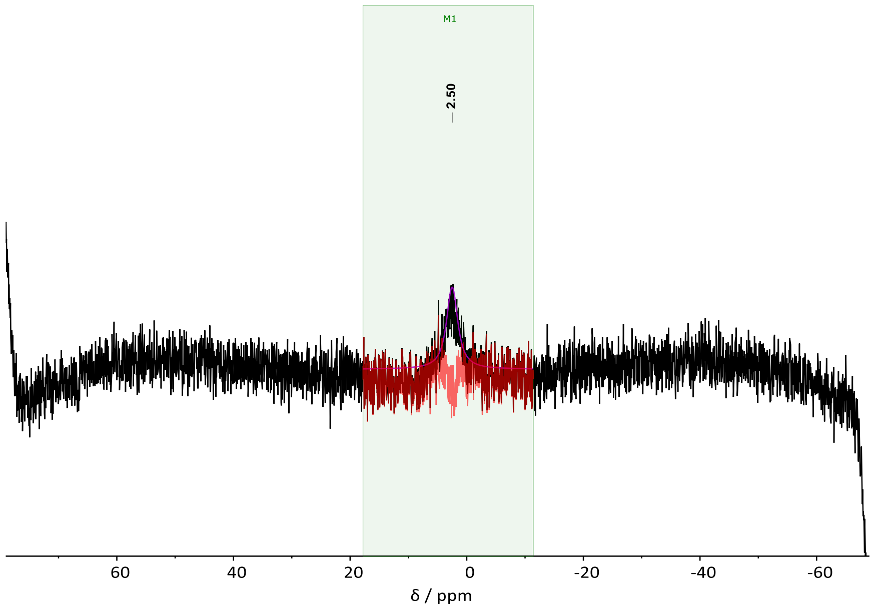

Figure S13. ⁹Be{¹H} NMR spectrum of [TP(iPr)]Be(nBu) in toluene-d₈

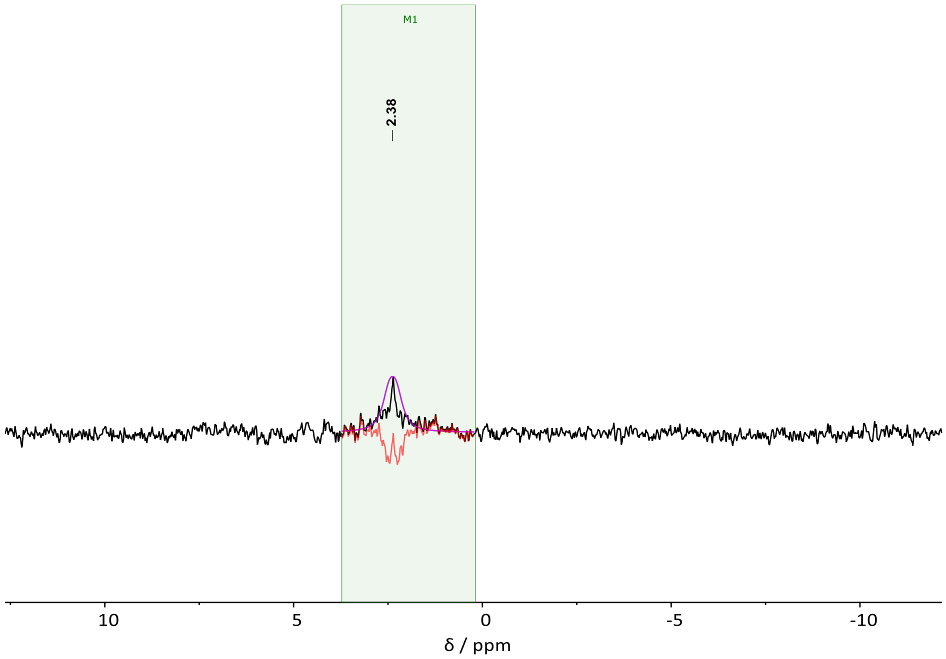

Figure S14. ⁹Be{¹H} NMR spectrum of [TP(iPr)]Be(nBu) in C₆D₆

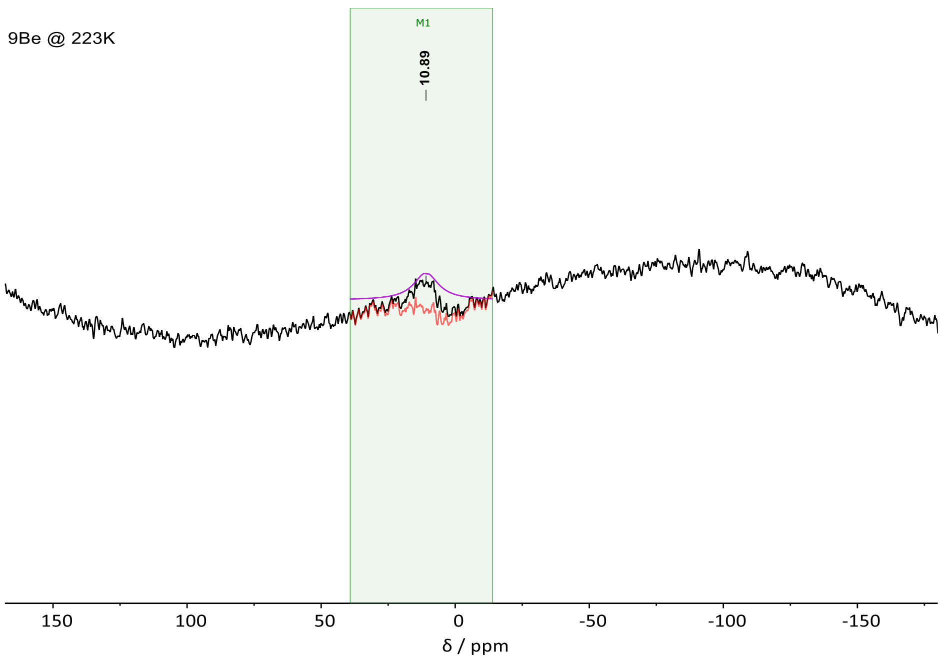

Figure S15. $^{9}Be\{^{1}H\}$ NMR spectrum of [TP(iPr)]Be(nBu) in toluene-d_8 at 223 K.

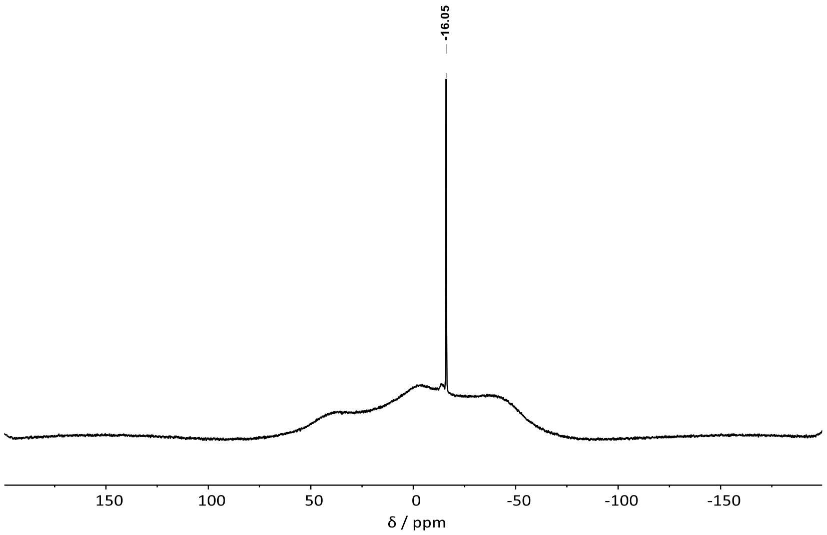

Figure S16. $^{11}B\{^{1}H\}$ NMR spectrum of [TP(iPr)]Be(nBu) in toluene-d_8.

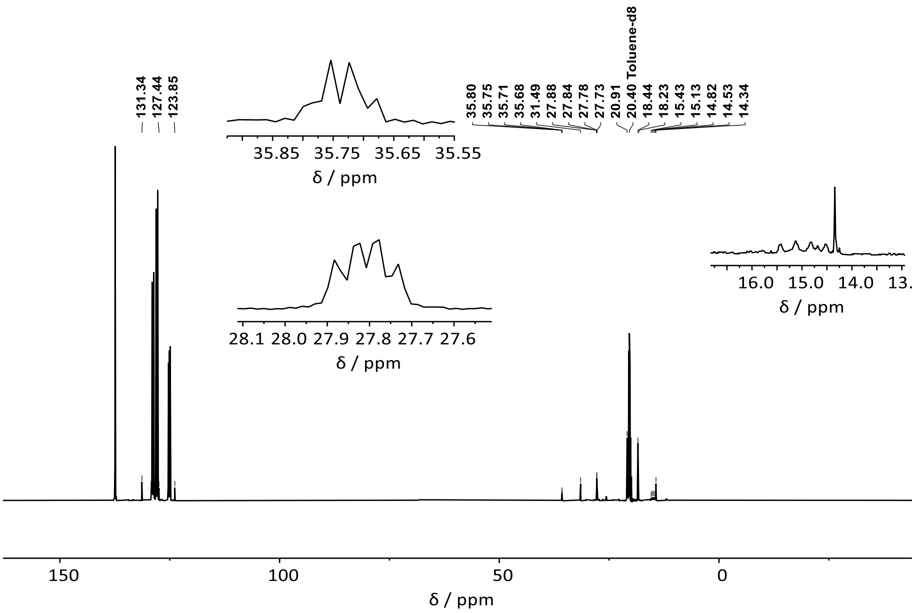

Figure S17. $^{13}C\{^{1}H\}$ NMR spectrum of [TP(iPr)]Be(nBu) in toluene-d_8.

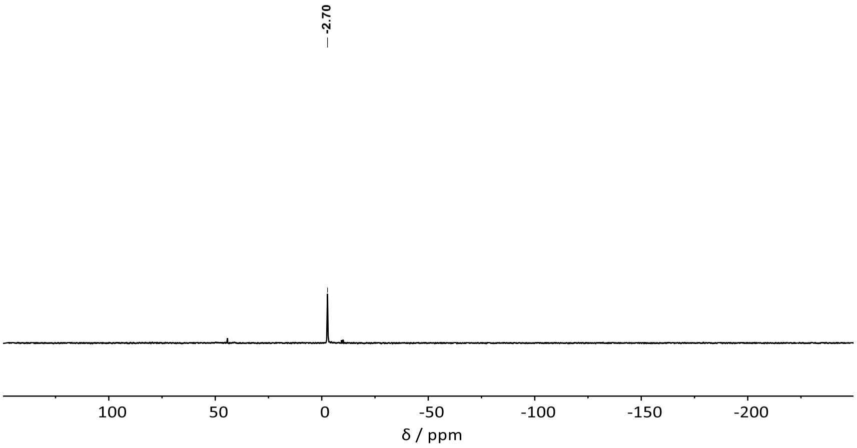

Figure S18. $^{31}P\{^{1}H\}$ NMR spectrum of [TP(iPr)]Be(nBu) in toluene-d_8.

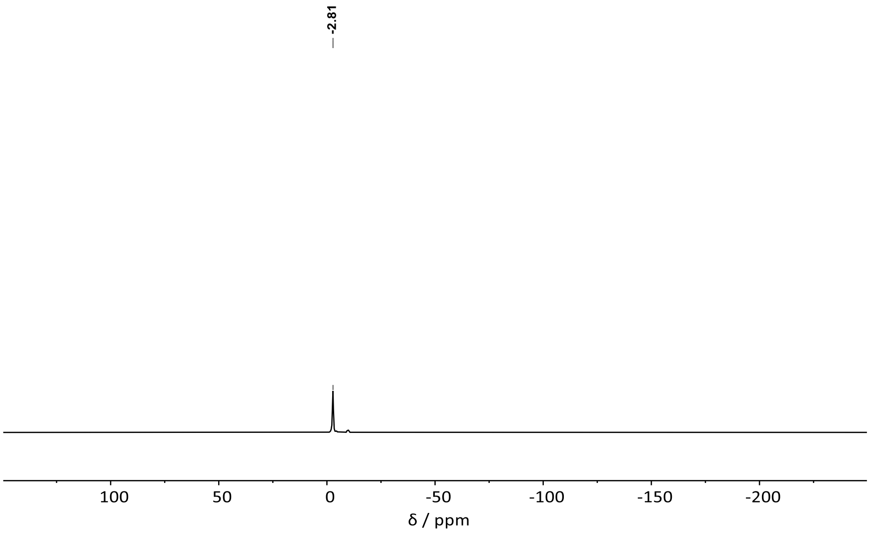

Figure S19. $^{31}P\{^1H\}$ NMR spectrum of [TP(iPr)]Be(nBu) in C_6D_6.

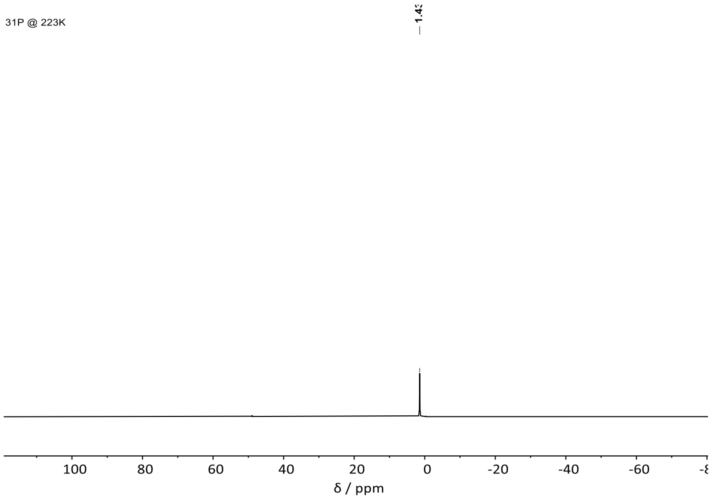

Figure S20. $^{31}P\{^1H\}$ NMR spectrum of [TP(iPr)]Be(nBu) in toluene-d_8 at 223 K.

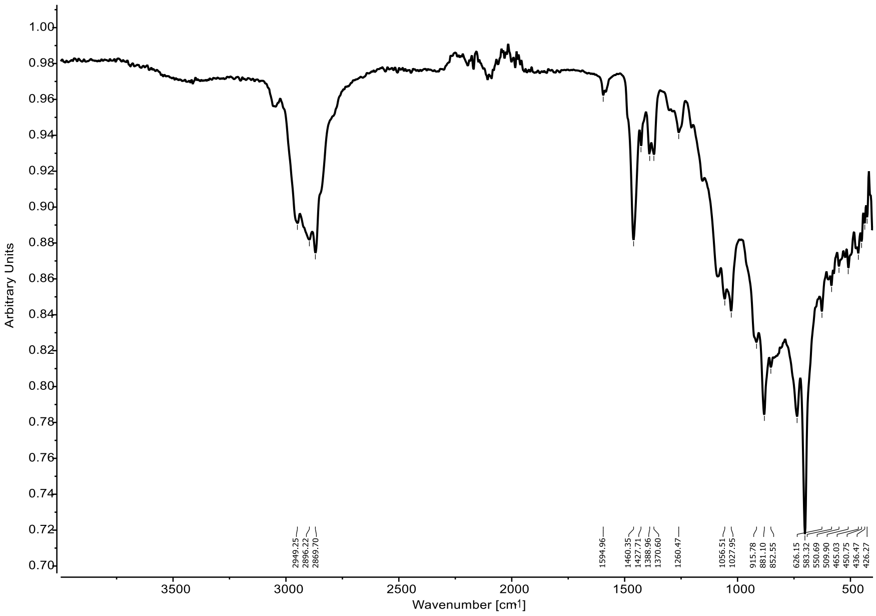

Figure S21. IR spectrum of [TP(iPr)]Be(nBu).

[TP(*i*Pr)O]BePh(**3d**)

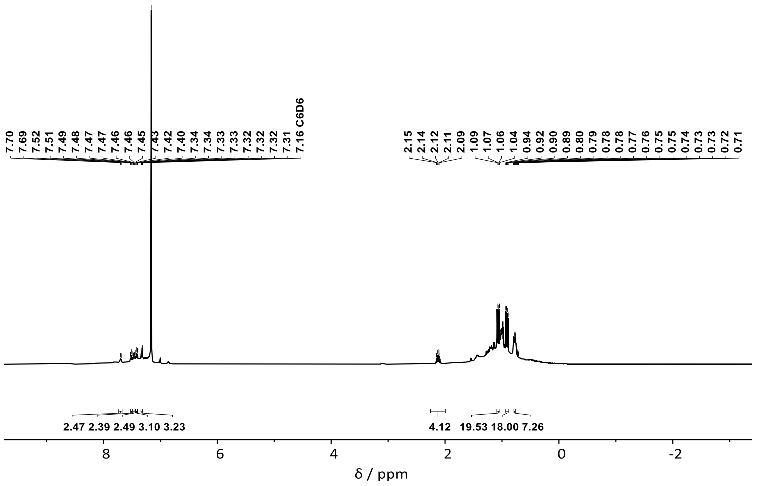

Figure S22. ^{1}H NMR spectrum of [TP(iPr)O]BePh in C_6D_6.

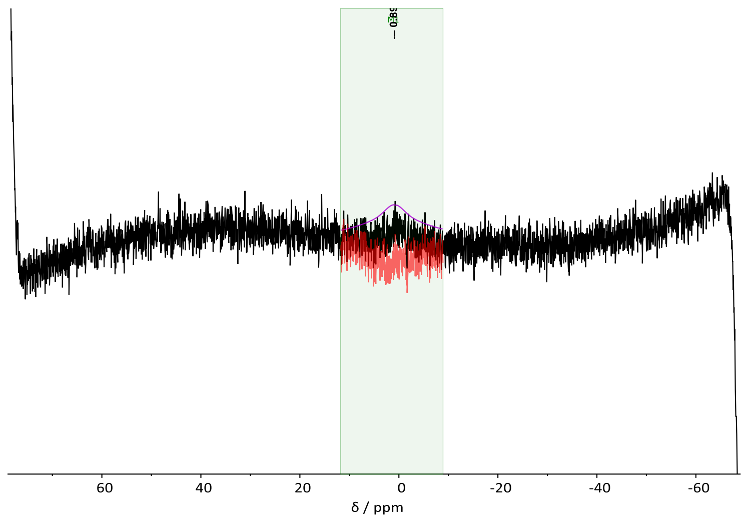

Figure S23. $^{9}Be\{^{1}H\}$ NMR spectrum of [TP(iPr)O]BePh in C_6D_6.

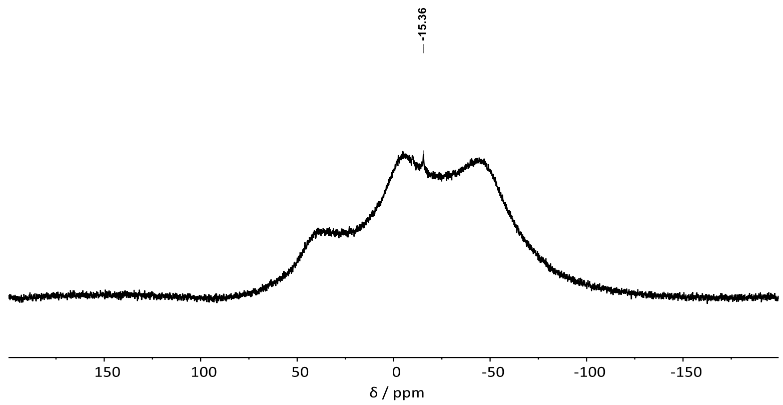

Figure S24. $^{11}B\{^{1}H\}$ NMR spectrum of [TP(iPr)O]BePh in C_6D_6.

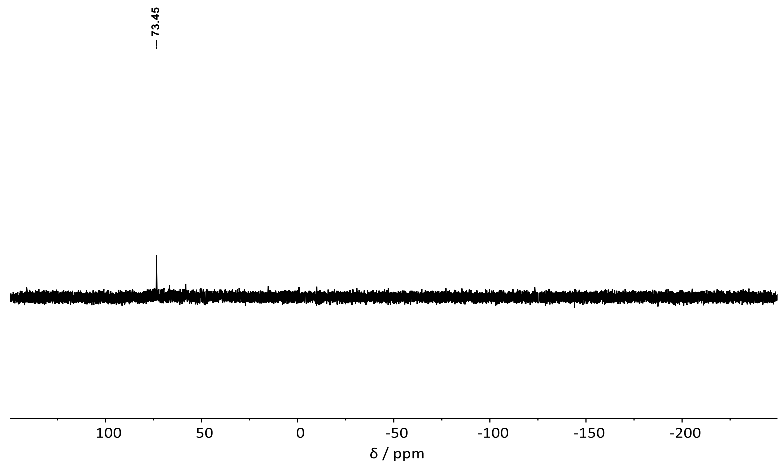

Figure S25. $^{31}P\{^{1}H\}$ NMR spectrum of [TP(iPr)O]BePh in C_6D_6.

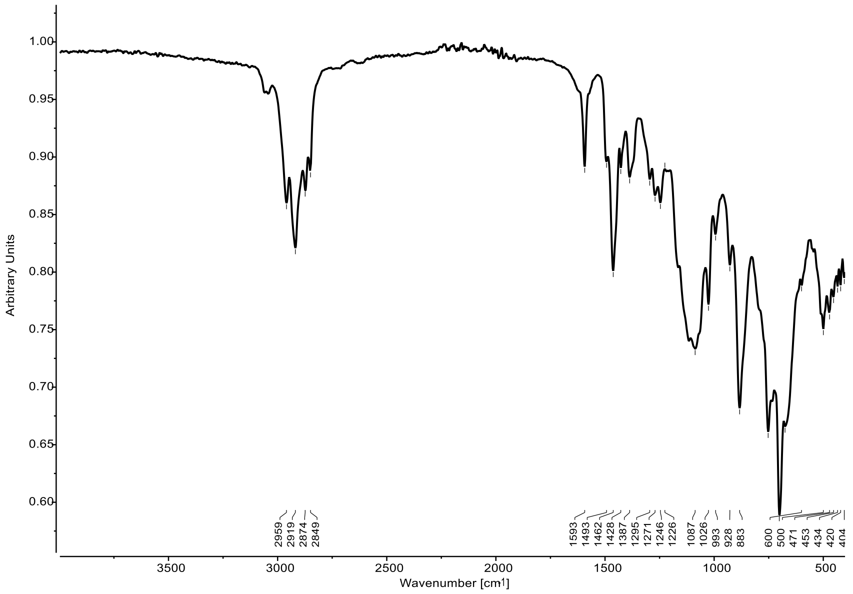

Figure S26. IR spectrum of [TP(iPr)O]BePh.

[TP(*i*Pr)*E*]BeCl (*E* = O, S, Se)

[TP(*i*Pr)O]BeCl (**3a**)

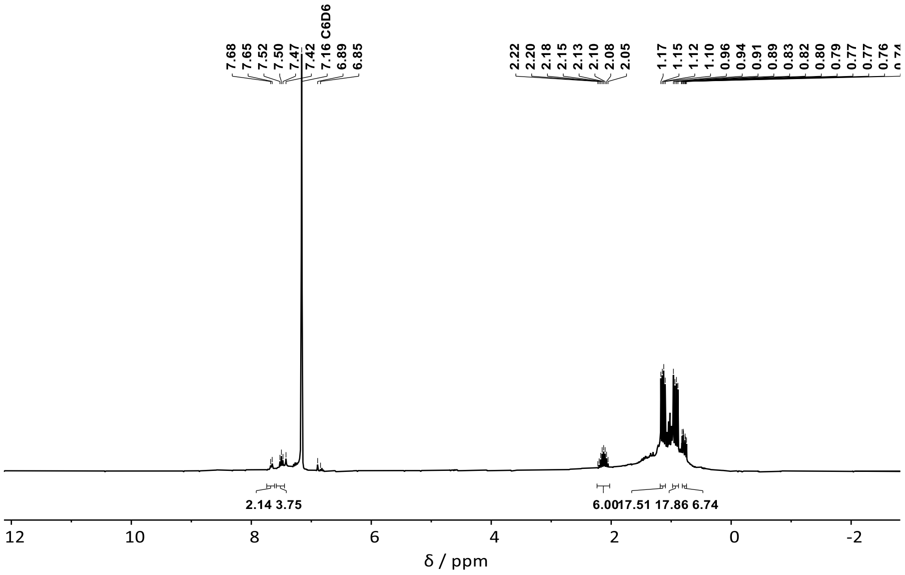

Figure S27. ^{1}H NMR spectrum of [TP(iPr)O]BeCl in C_6D_6.

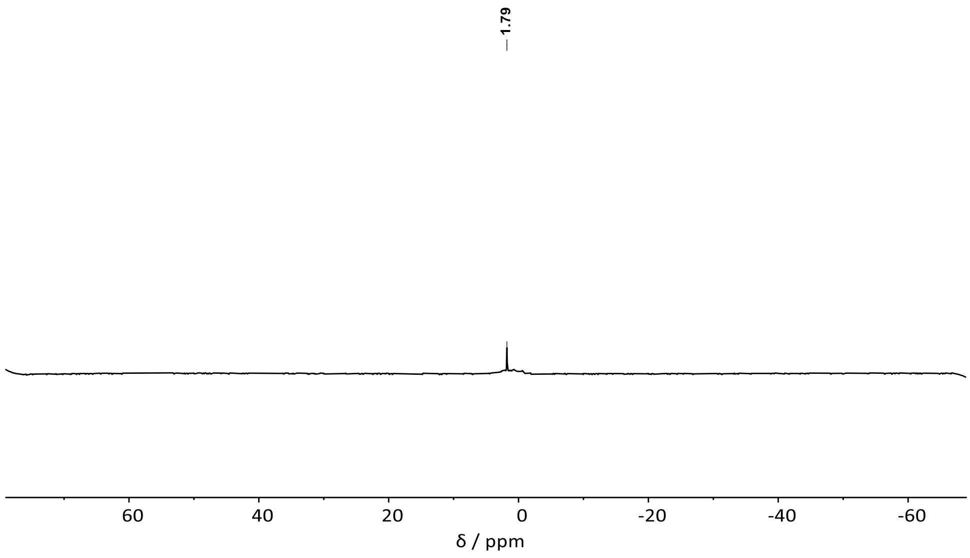

Figure S28. $^{9}Be\{^{1}H\}$ NMR spectrum of [TP(iPr)O]BeCl in C_6D_6.

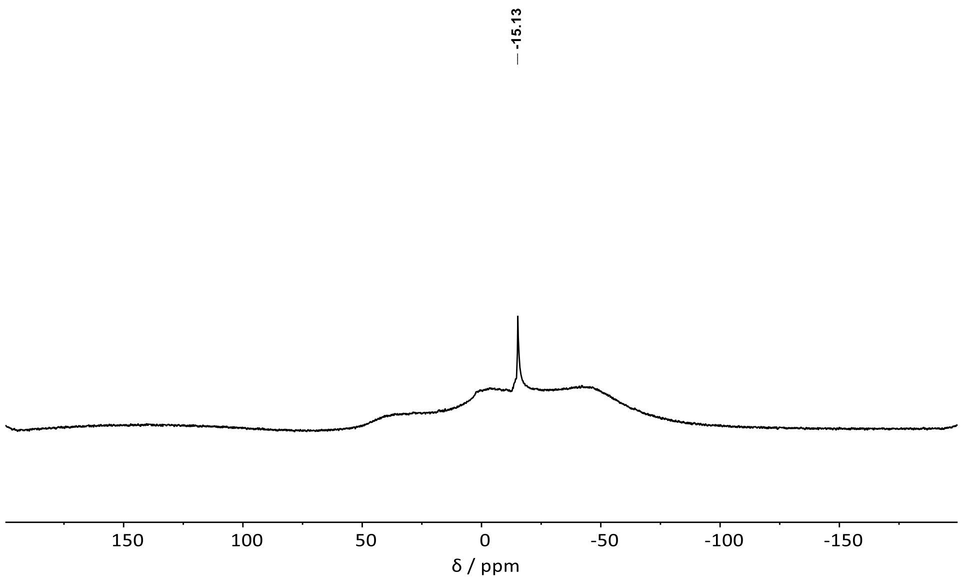

Figure S29. $^{11}B\{^{1}H\}$ NMR spectrum of [TP(iPr)O]BeCl in C_6D_6.

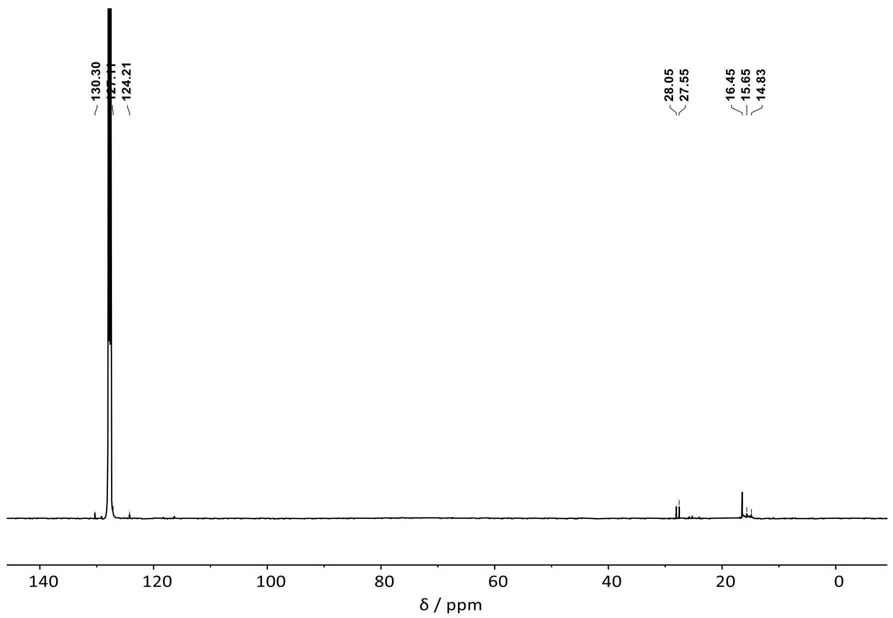

Figure S30. $^{13}C\{^{1}H\}$ *NMR spectrum of [TP(iPr)O]BeCl in* C_6D_6.

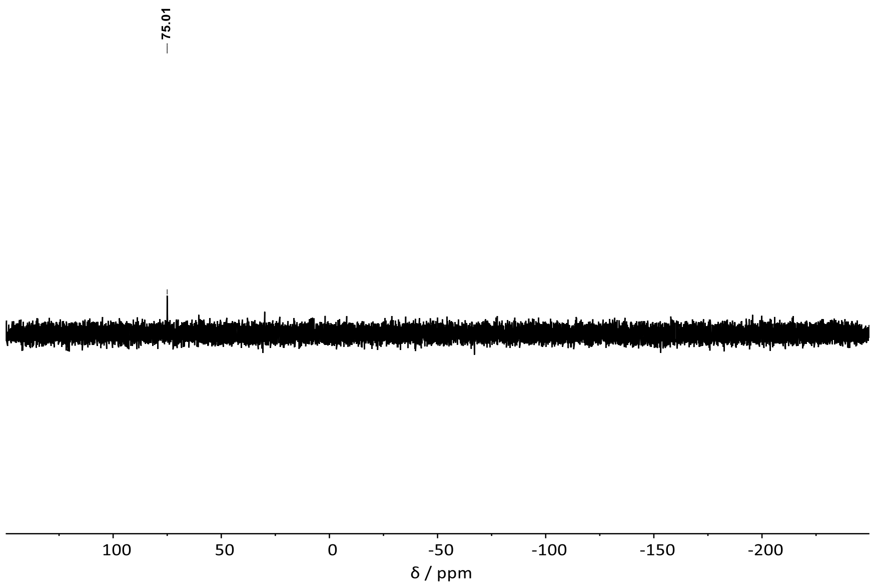

Figure S31. $^{31}P\{^{1}H\}$ *NMR spectrum of [TP(iPr)O]BeCl in* C_6D_6.

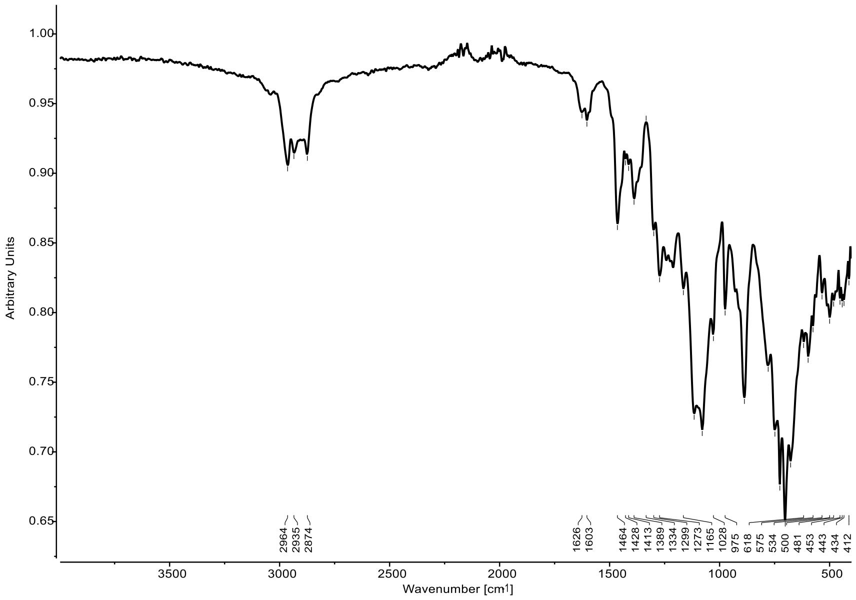

Figure S32. IR spectrum of [TP(iPr)O]BeCl.

[TP(*i*Pr)S]BeCl (**3b**)

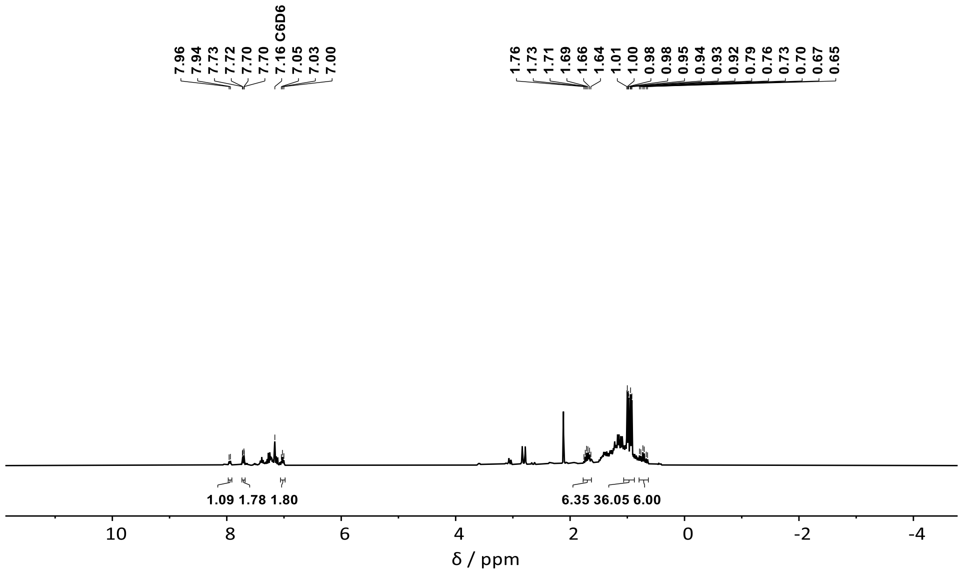

Figure S33. ¹H NMR spectrum of [TP(iPr)S]BeCl in C_6D_6.

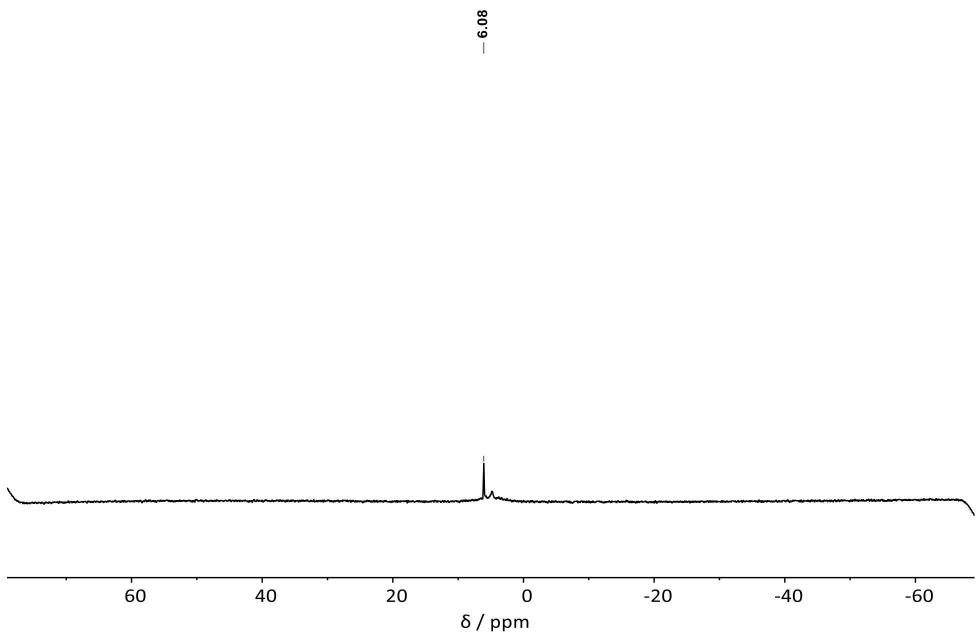

Figure S34. $^{9}Be\{^{1}H\}$ NMR spectrum of [TP(iPr)S]BeCl in C_6D_6.

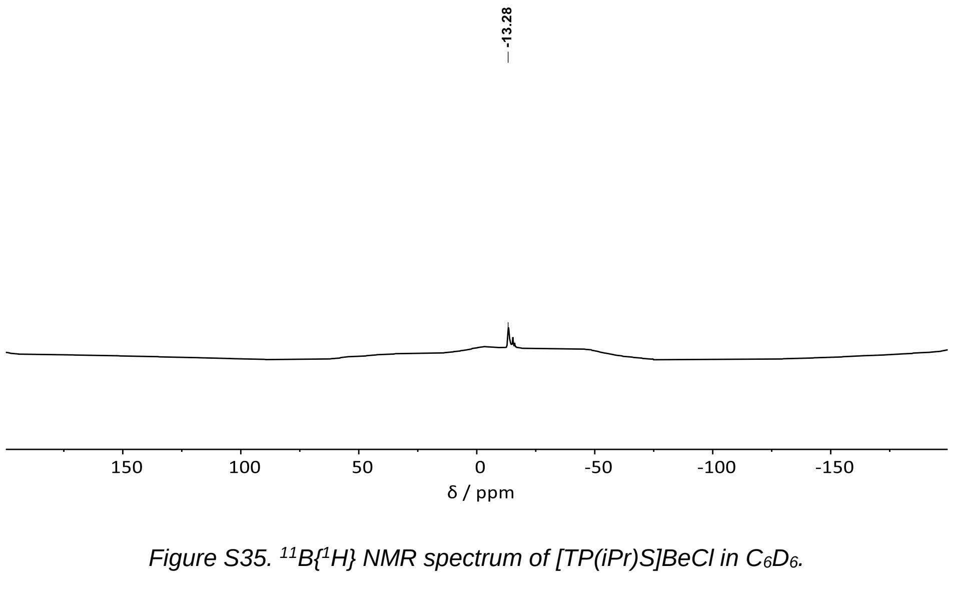

Figure S35. $^{11}B\{^{1}H\}$ NMR spectrum of [TP(iPr)S]BeCl in C_6D_6.

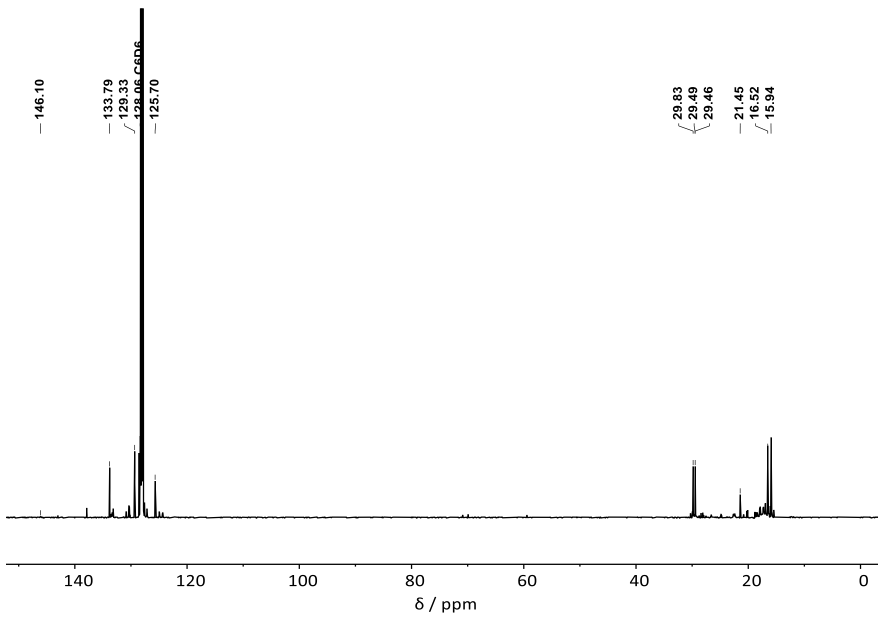

Figure S36. $^{13}C\{^{1}H\}$ NMR spectrum of [TP(iPr)S]BeCl in C_6D_6.

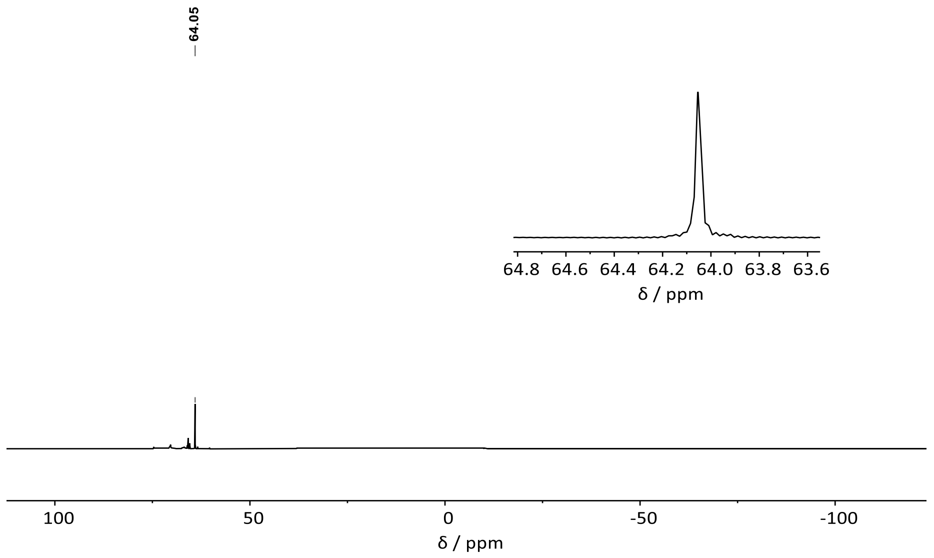

Figure S37. $^{31}P\{^{1}H\}$ NMR spectrum of [TP(iPr)S]BeCl in C_6D_6.

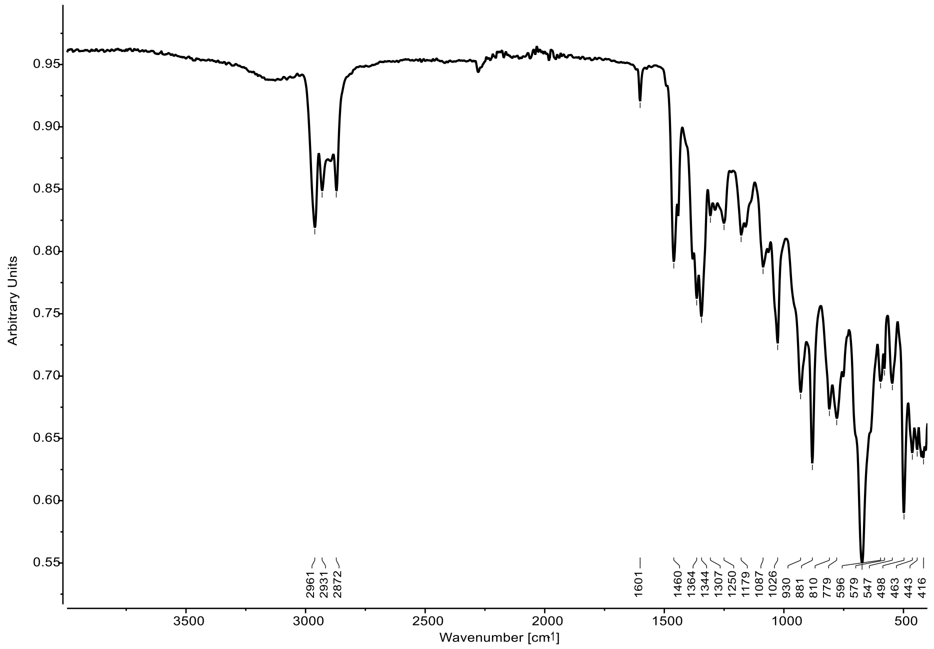

Figure S38. IR spectrum of [TP(iPr)S]BeCl.

[TP(*i*Pr)Se]BeCl (**3c**)

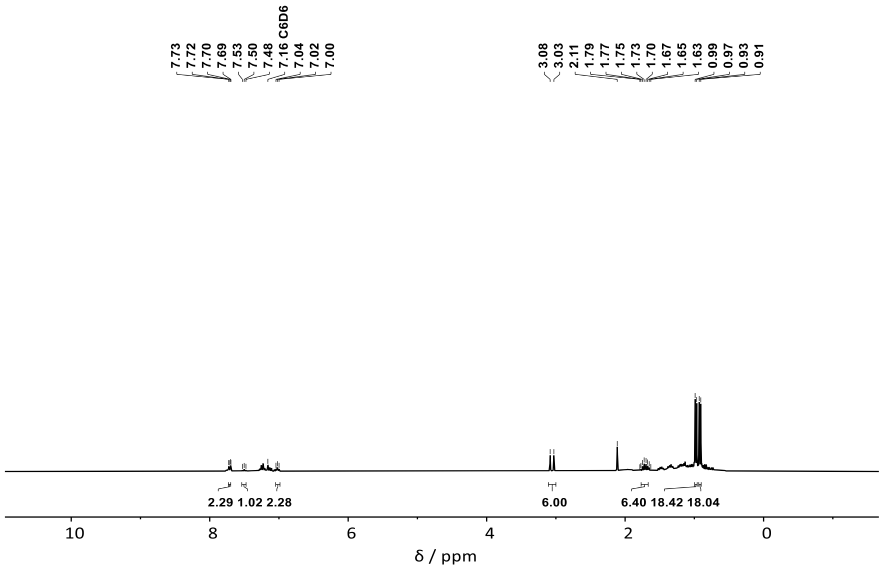

Figure S39. ^{1}H NMR spectrum of [TP(iPr)Se]BeCl in C_6D_6.

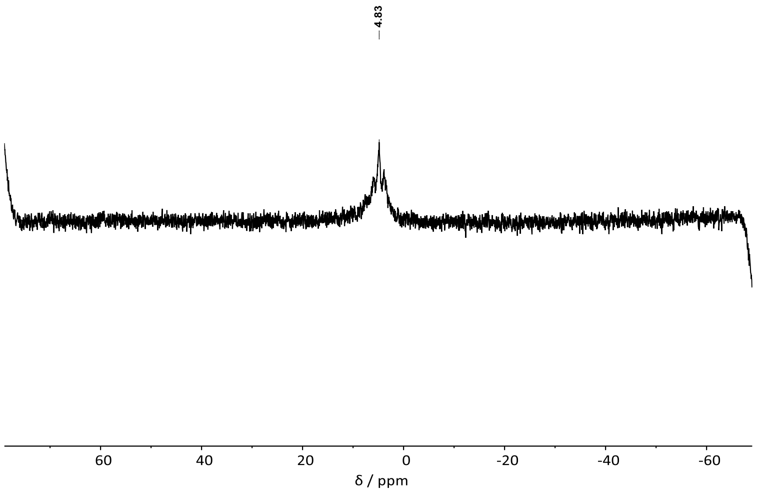

Figure S40. $^{9}Be\{^{1}H\}$ NMR spectrum of [TP(iPr)Se]BeCl in C_6D_6.

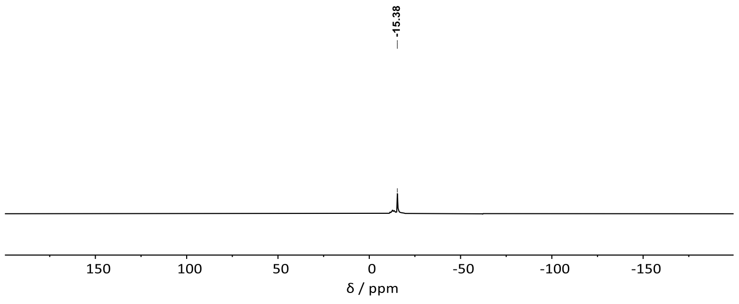

Figure S41. $^{11}B\{^{1}H\}$ NMR spectrum of [TP(iPr)Se]BeCl in C_6D_6.

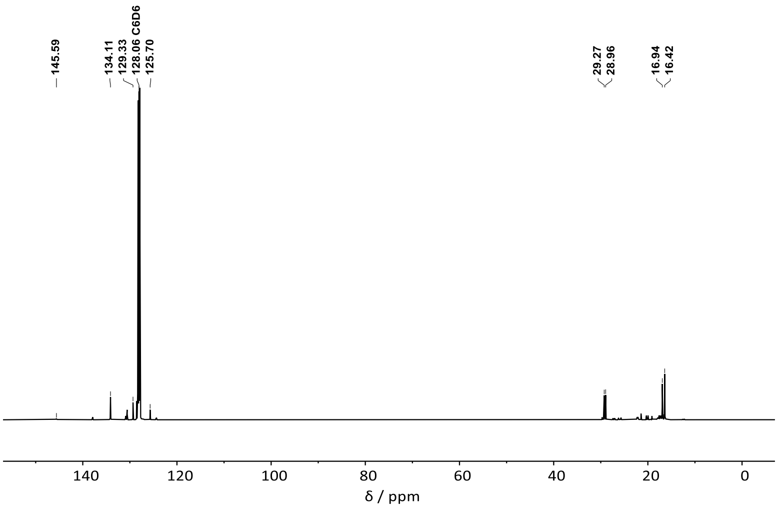

Figure S42. $^{13}C\{^{1}H\}$ NMR spectrum of [TP(iPr)Se]BeCl in C_6D_6.

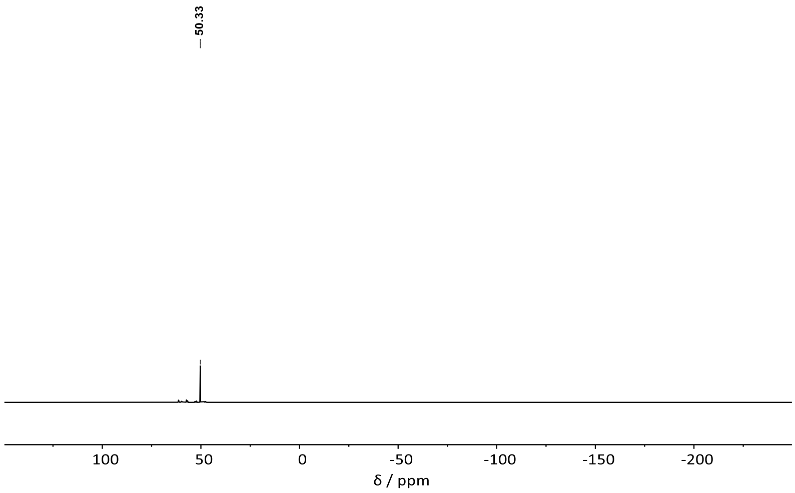

Figure S43. $^{31}P\{^1H\}$ NMR spectrum of [TP(iPr)Se]BeCl in C_6D_6.

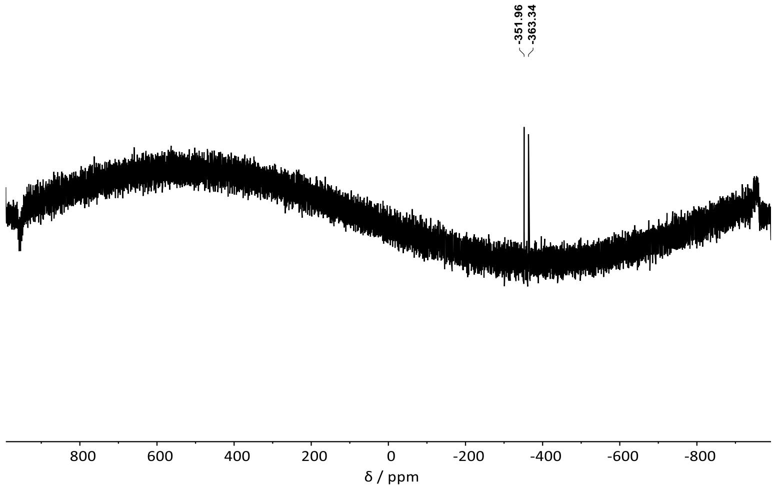

Figure S44. ^{77}Se NMR spectrum of [TP(iPr)Se]BeCl in C_6D_6.

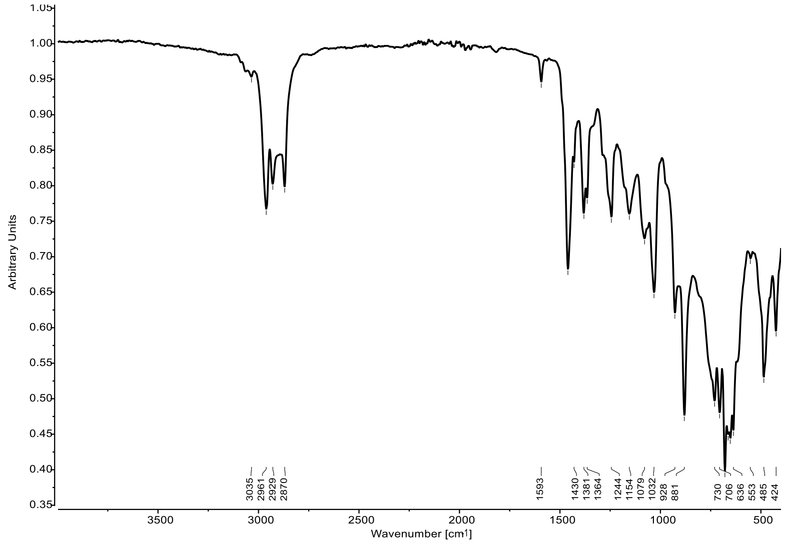

Figure S45. IR spectrum of [TP(iPr)Se]BeCl.

[(Cl)Be{κ–Se(*i*Pr)$_2$PCH$_2$}μ–Se]$_2$ (5)

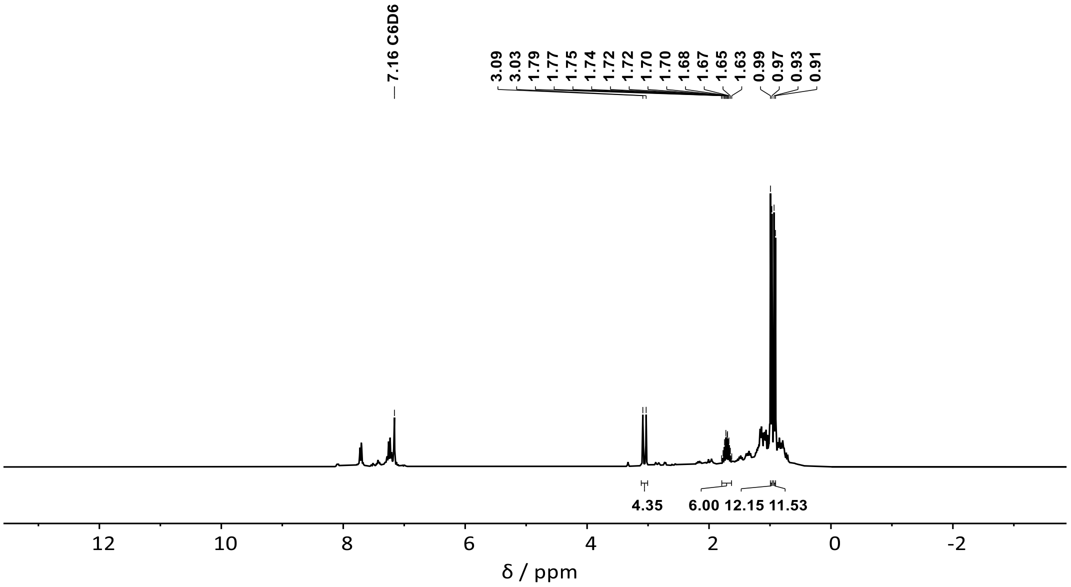

*Figure S46. ^{1}H NMR spectrum of **5** in C_6D_6. A small amounts of [TP(iPr)Se]BeCl is also present.*

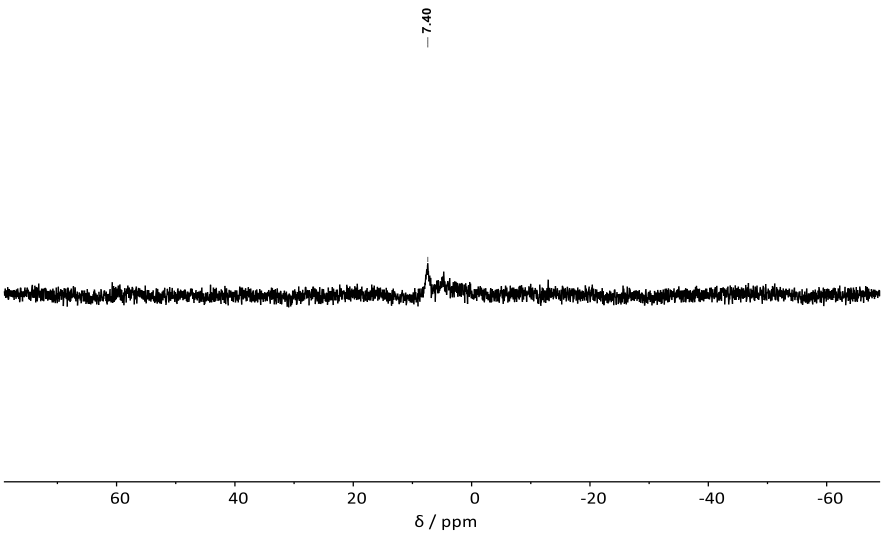

*Figure S47. ^{9}Be{^{1}H} NMR spectrum of **5** in C_6D_6.*

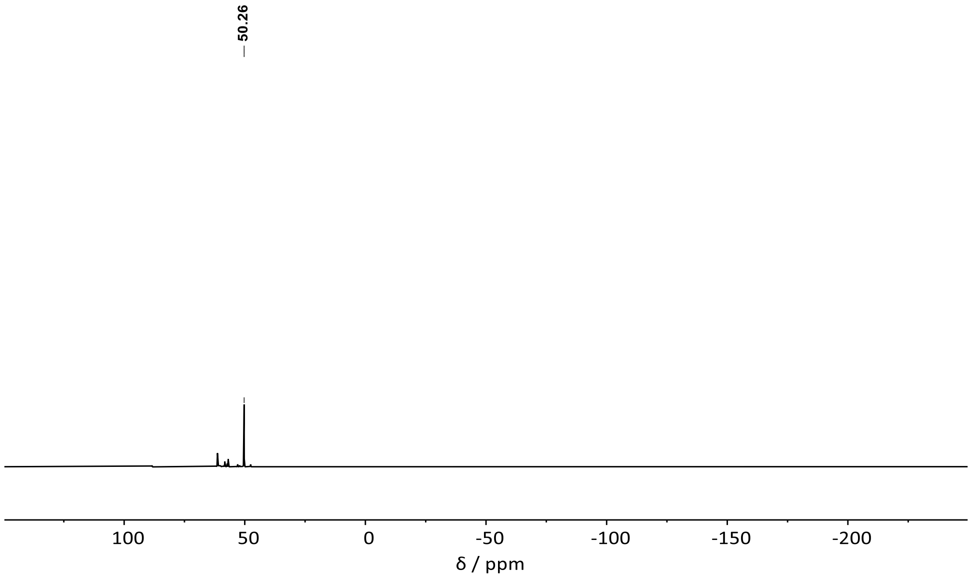

*Figure S48. $^{31}P\{^{1}H\}$ NMR spectrum of **5** in C_6D_6.*

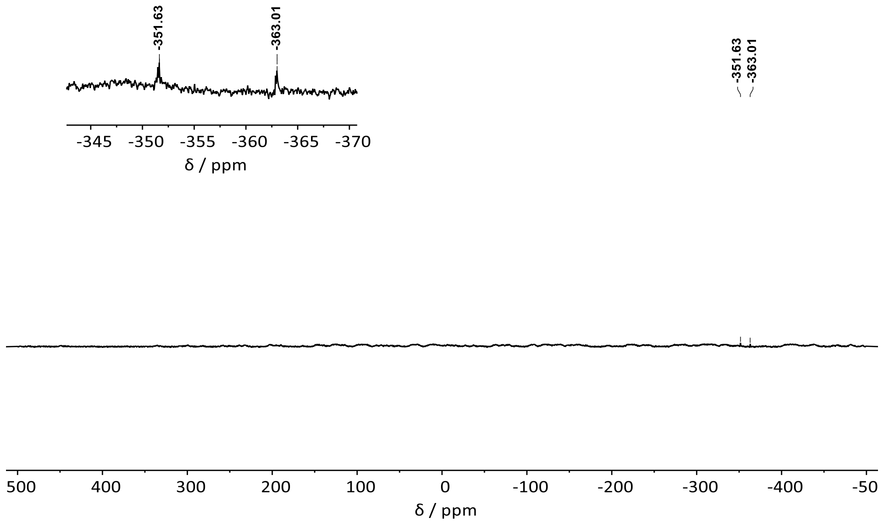

*Figure S49. ^{77}Se NMR spectrum of **5** in C_6D_6.*

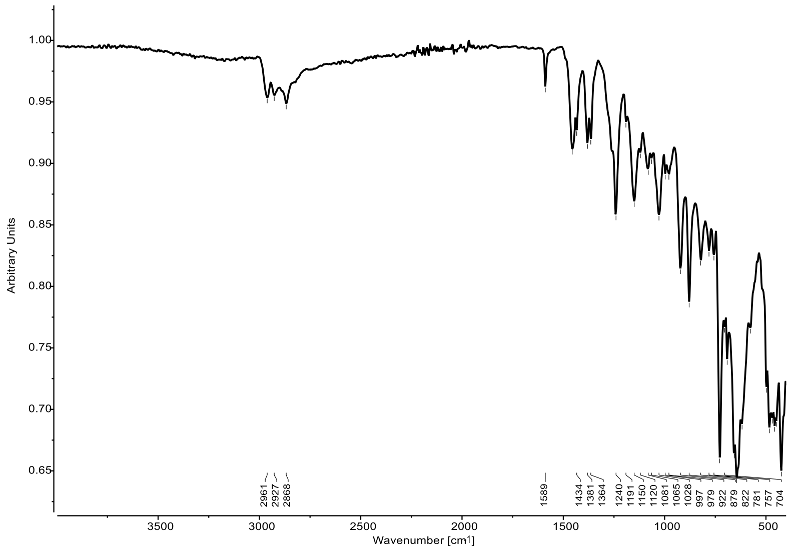

*Figure S50. IR spectrum of **5**.*

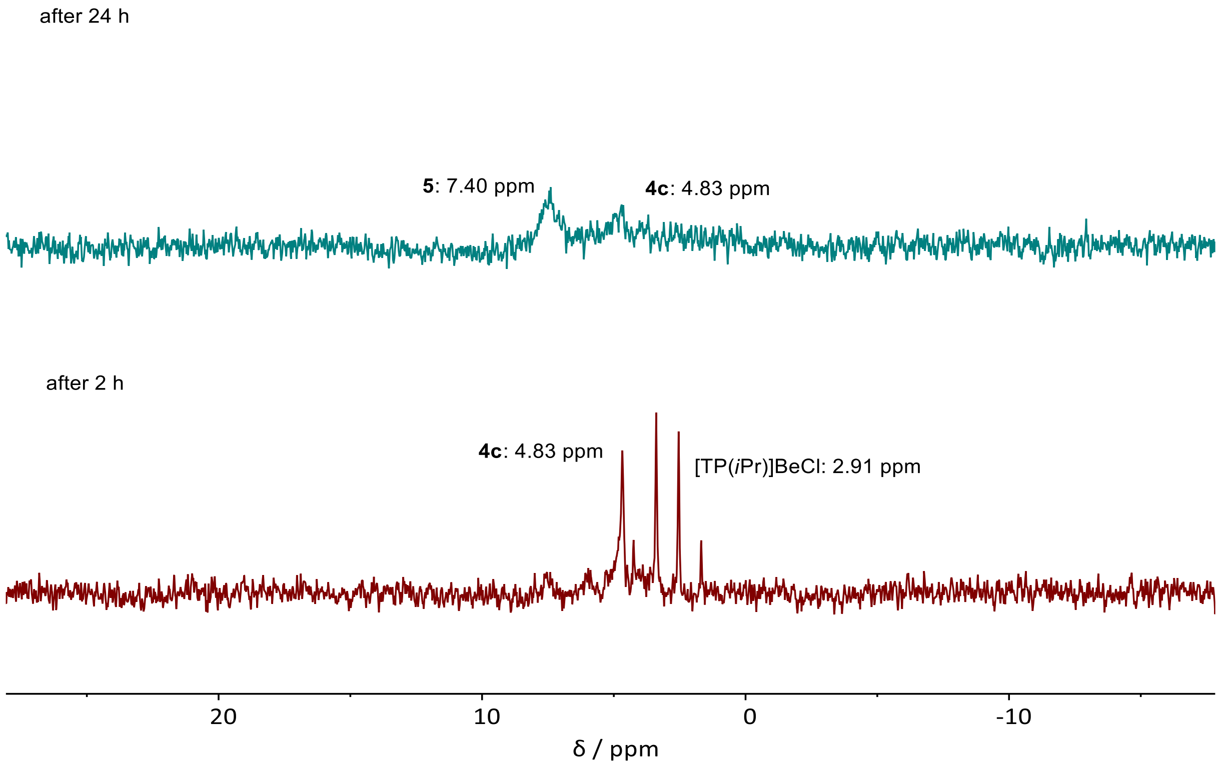

Figure S51: ^{9}Be{^{1}H} NMR reaction control spectra of [TP(iPr)Se]BeCl and excess selenium at different reaction times.

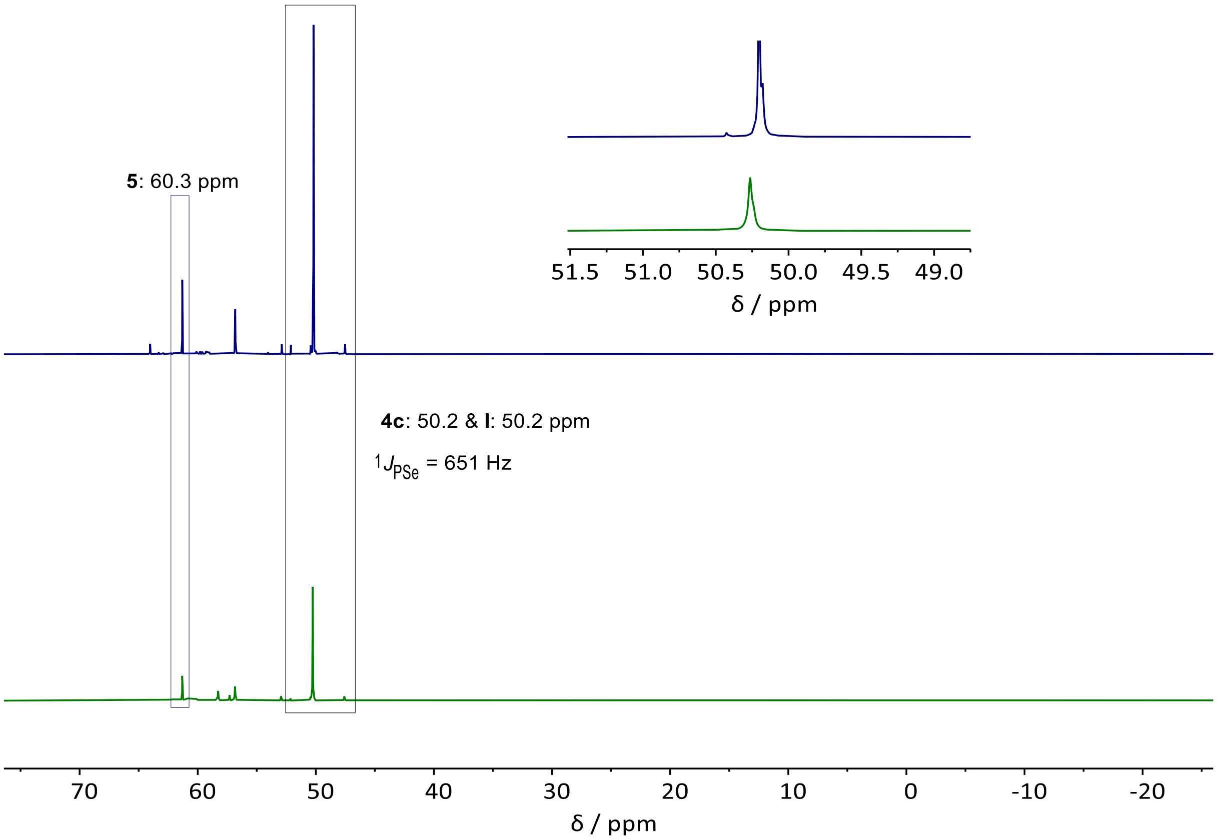

Figure S52: $^{31}P\{^1H\}$ NMR reaction control spectra of [TP(iPr)Se]BeCl and excess selenium at different reaction times.

[Ph$_2$B(µ-P(*i*Pr)$_2$CH$_2$)]$_2$ (6)

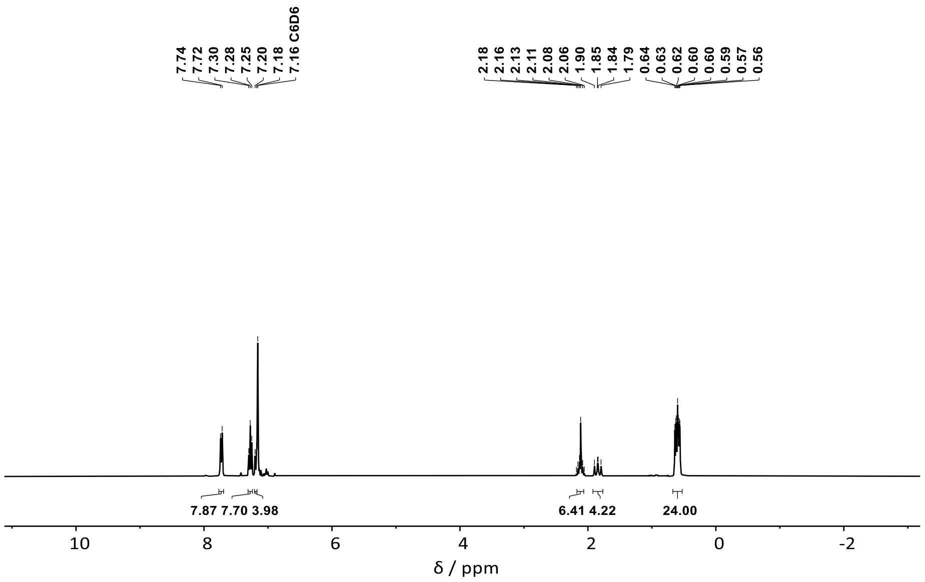

*Figure S53. ^1H NMR spectrum of **6** in C_6D_6.*

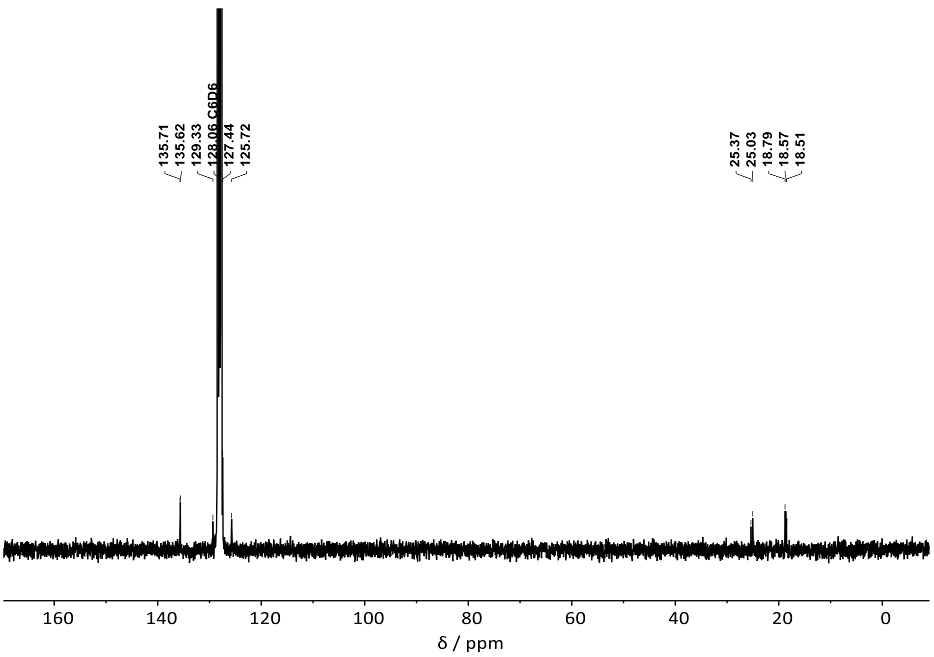

*Figure S54. $^{13}C\{^1H\}$ NMR spectrum of **6** in C_6D_6.*

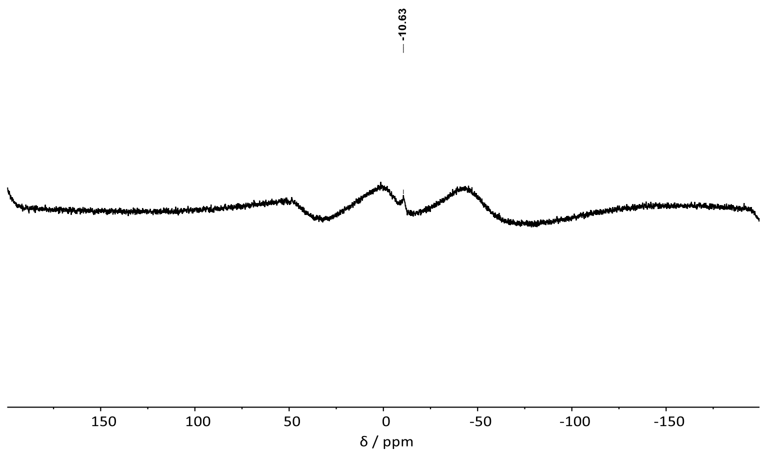

*Figure S55. $^{11}B\{^1H\}$ NMR spectrum of **6** in C_6D_6.*

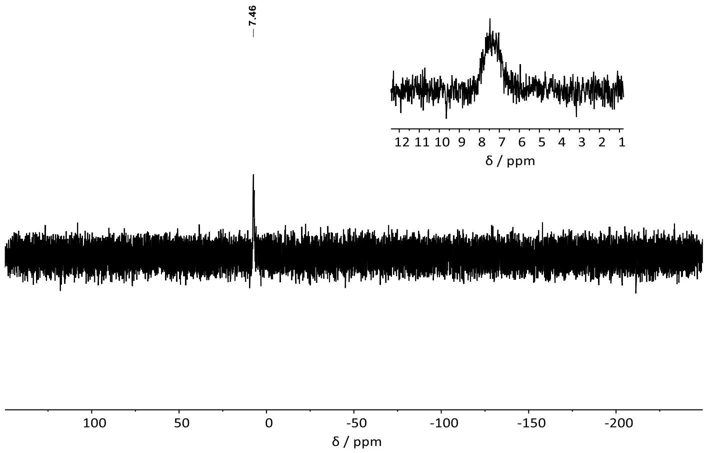

*Figure S56. $^{31}P\{^{1}H\}$ NMR spectrum of **6** in C_6D_6.*

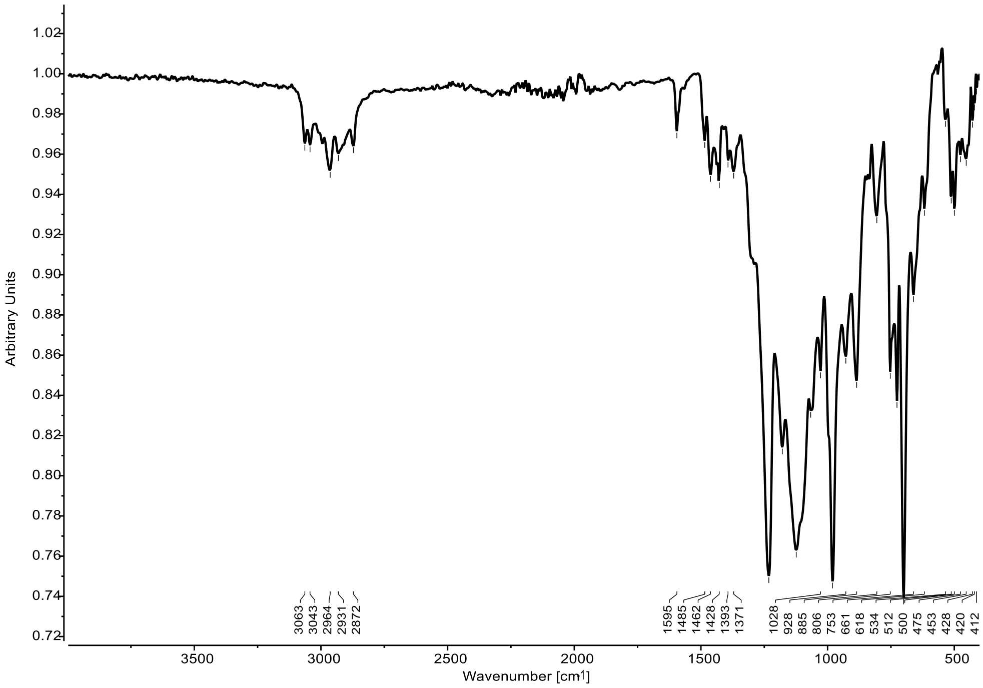

*Figure S57. IR spectrum of **6**.*

[PhB{CH_2P(*i*Pr)$_2$}μ-P(*i*Pr)$_2$$CH_2$]$_2$ (2)

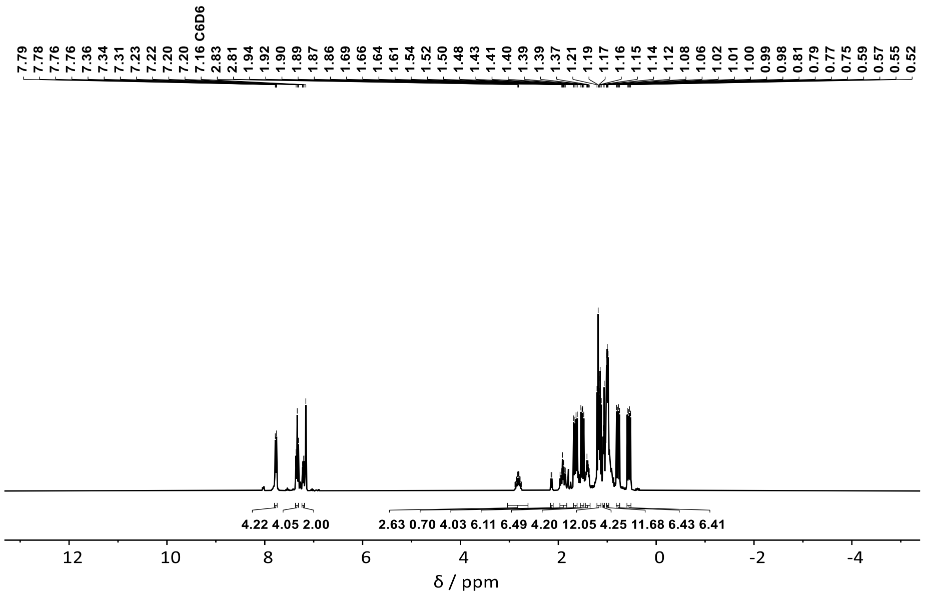

*Figure S58. ^{1}H NMR spectrum of **2** in C_6D_6. A small amount of [TP(iPr)]BeCl is also present.*

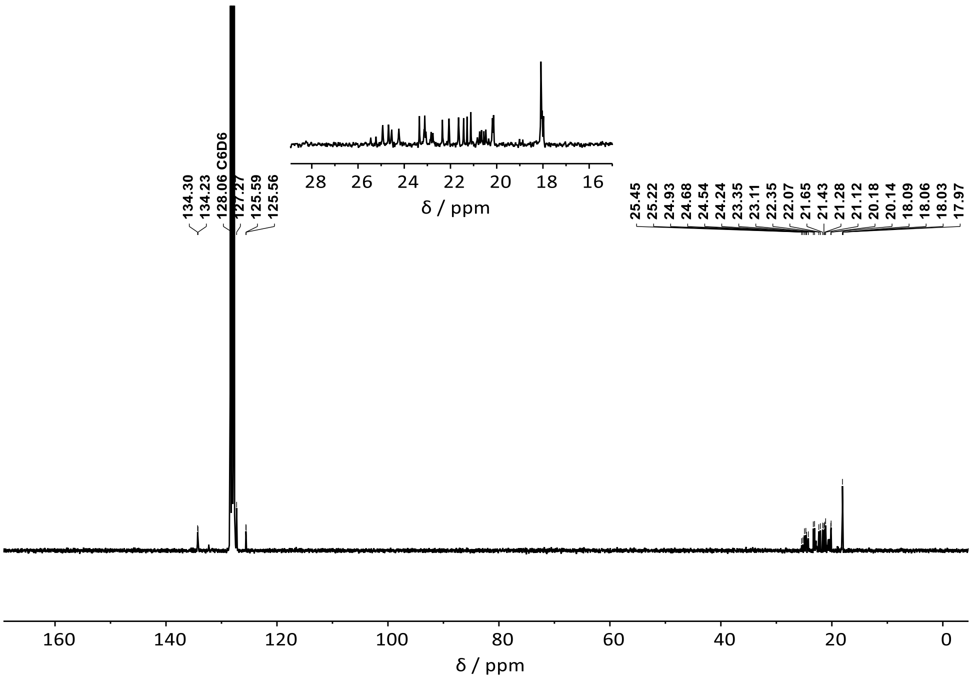

*Figure S59. ^{13}C{^{1}H} NMR spectrum of **2** in C_6D_6.*

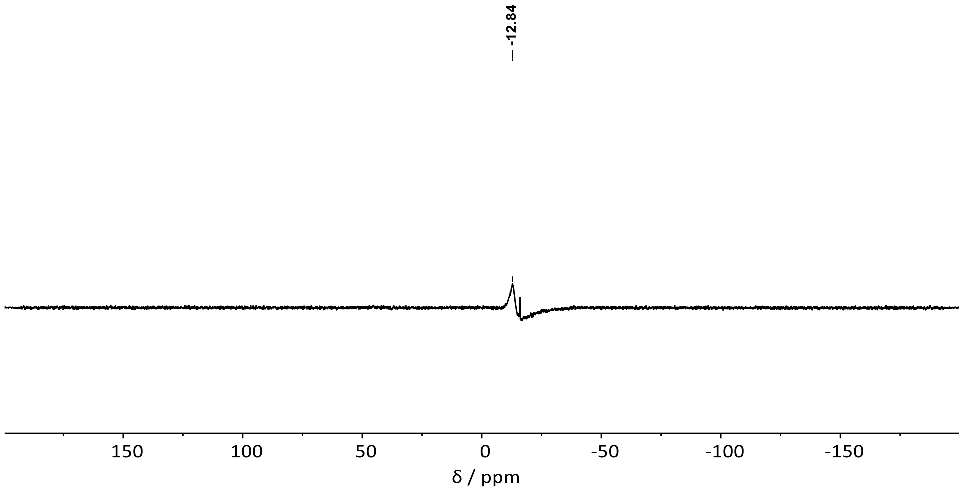

*Figure S60. $^{11}B\{^1H\}$ NMR spectrum of **5** in C_6D_6. A small amount of [TP(iPr)]BeCl is also present.*

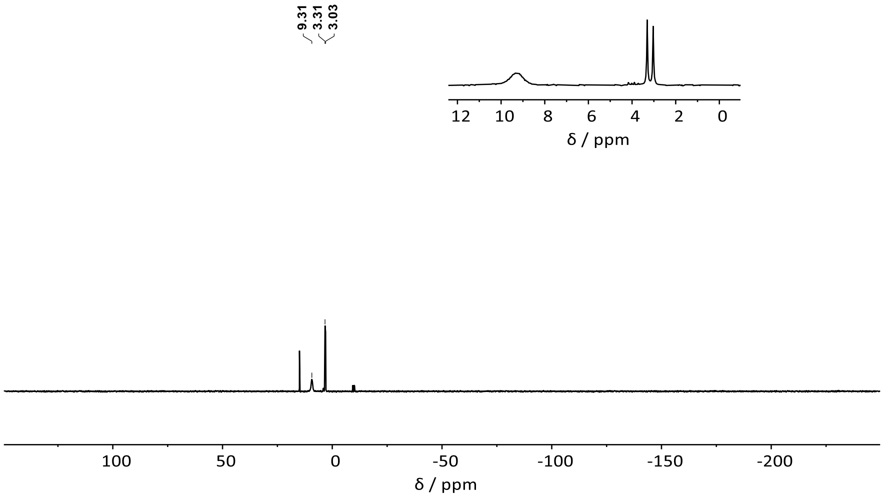

*Figure S61. $^{31}P\{^1H\}$ NMR spectrum of **2** in C_6D_6. A small amount of [TP(iPr)]BeCl is also present.*

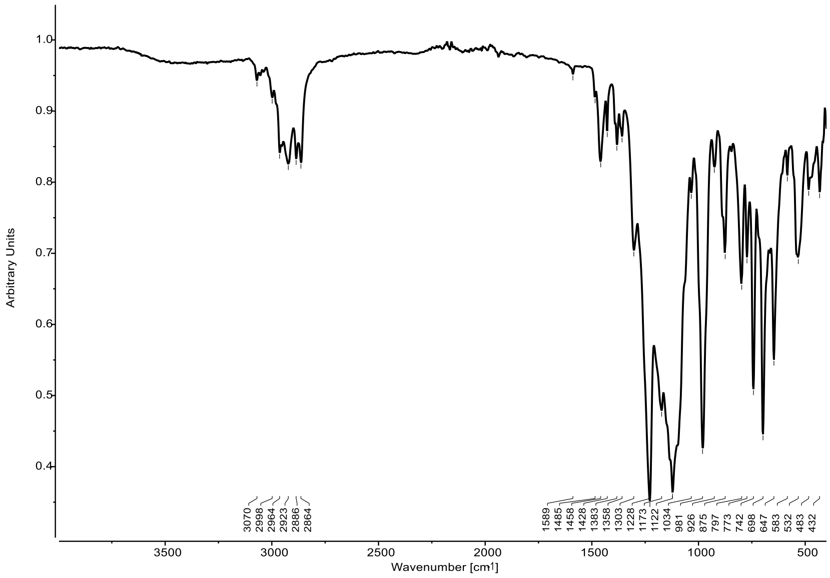

*Figure S62. IR spectrum of **2**.*

[Be(*R’*)(CH₂P(*i*Pr)₂)] (*R’* = Cp*, Cp)

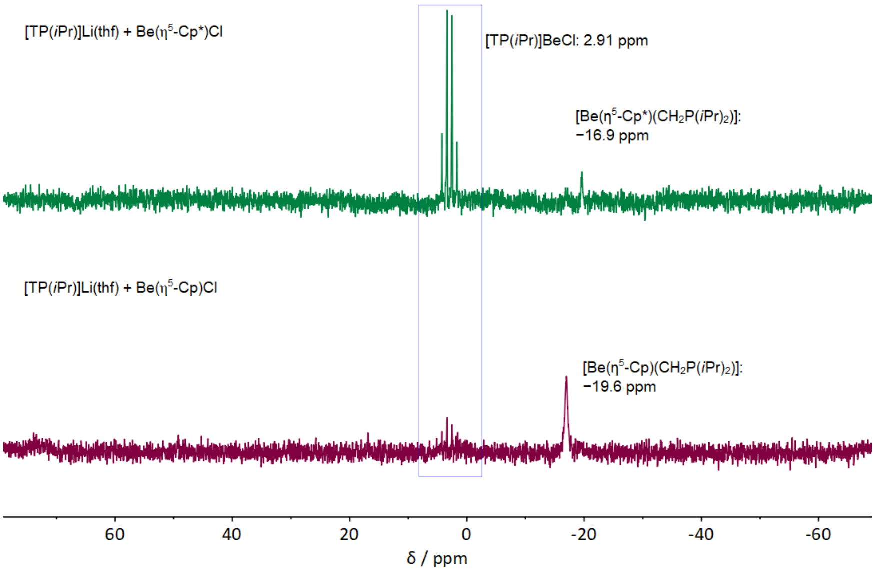

Figure S63. ^{9}Be NMR reaction spectra of [TP(iPr)]Li(thf) with Be(η^5-R’)Cl in benzene.

[Be(η^5-Cp)(CH_2P(*i*Pr)$_2$)] (**3a**)

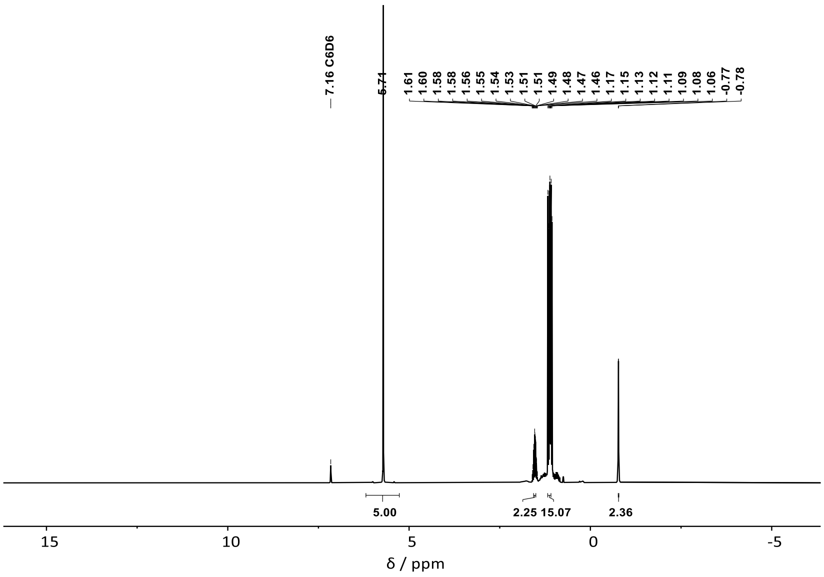

Figure S64. ^{1}H NMR spectrum of [Be(η^5-Cp)(CH_2P(iPr)$_2$)] in C_6D_6.

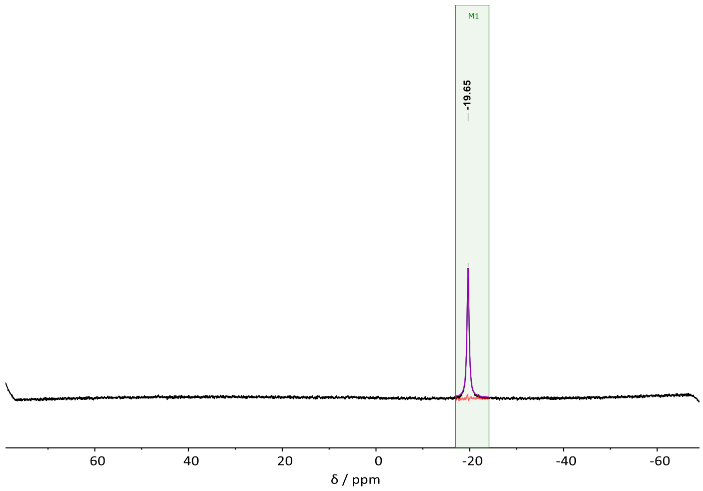

Figure S65. ^{9}Be{^{1}H} NMR spectrum of [Be(η^5-Cp)(CH_2P(iPr)$_2$)] in C_6D_6.

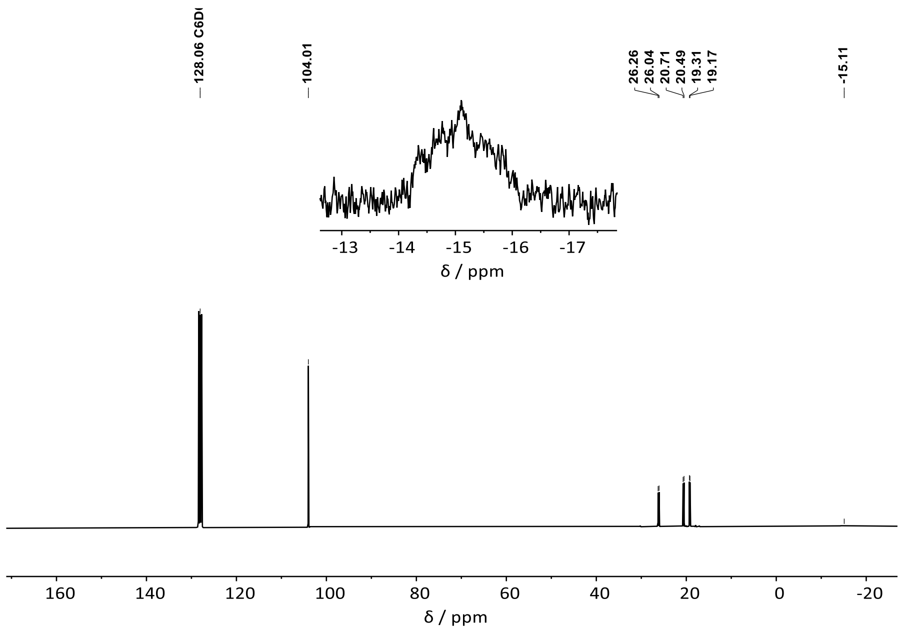

Figure S66. $^{13}C\{^1H\}$ NMR spectrum of $[Be(\eta^5\text{-}Cp)(CH_2P(iPr)_2)]$ in C_6D_6.

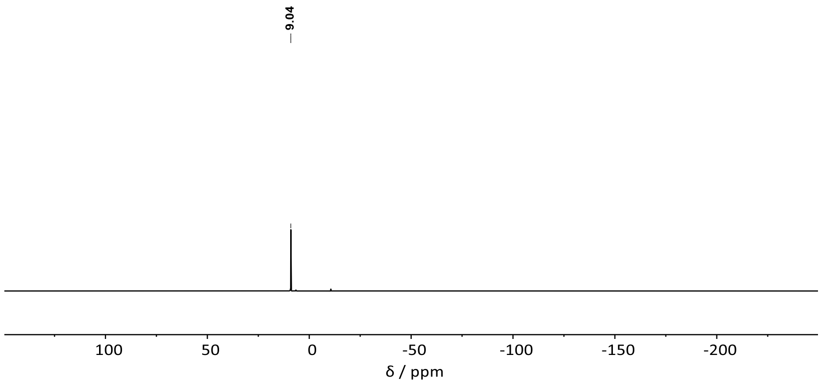

Figure S67. $^{31}P\{^1H\}$ NMR spectrum of $[Be(\eta^5\text{-}Cp)(CH_2P(iPr)_2)]$ in C_6D_6.

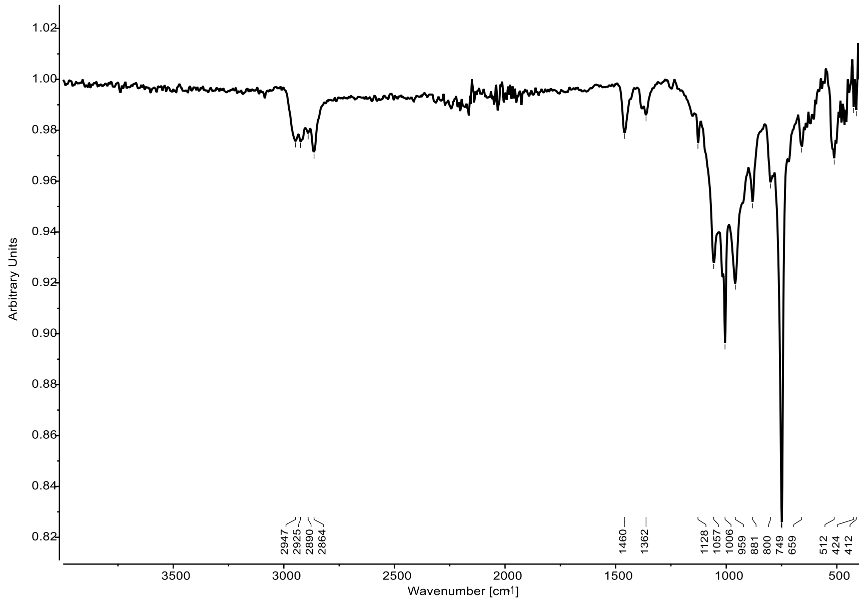

Figure S68. IR spectrum of [Be(η^5-Cp)($CH_2P(iPr)_2$)].

[Be(η^5-Cp*)(CH_2P(*i*Pr)$_2$)] (**3b**)

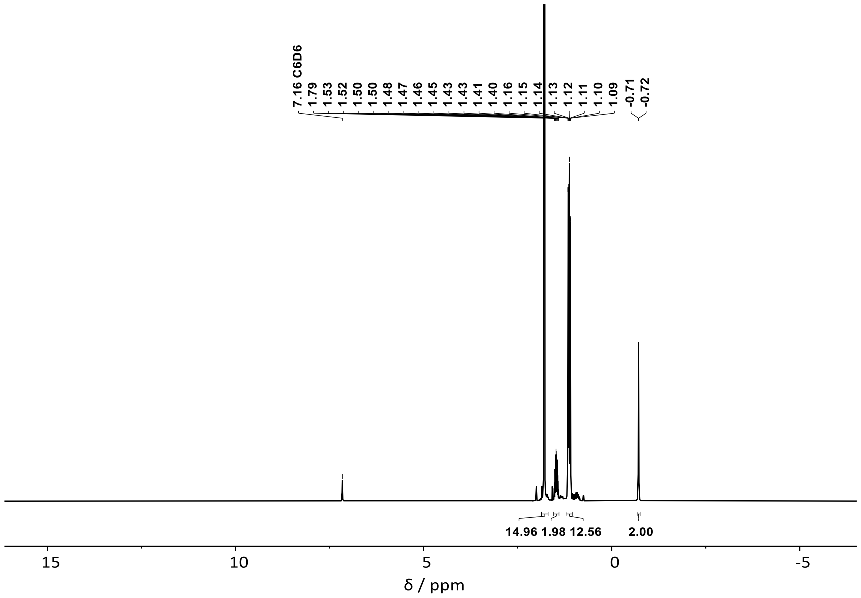

Figure S69. ^1H NMR spectrum of [Be(η^5-Cp)($CH_2P(iPr)_2$)] in C_6D_6.*

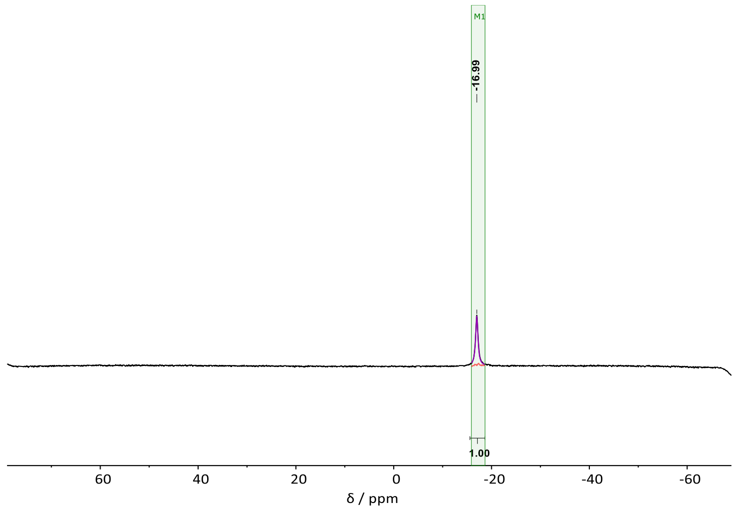

Figure S70. $^{9}Be\{^{1}H\}$ NMR spectrum of [Be(η^5-Cp)($CH_2P(iPr)_2$) in C_6D_6.*

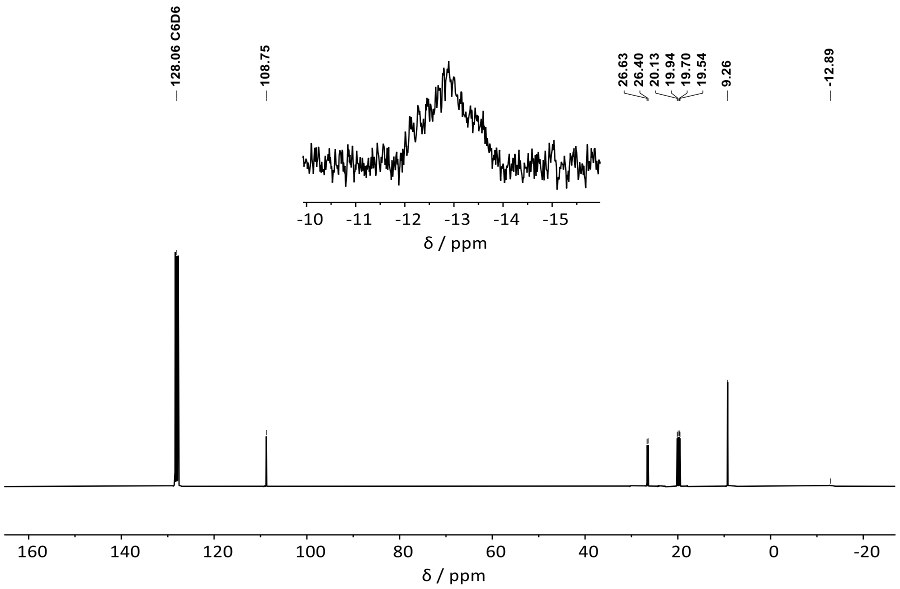

Figure S71. $^{13}C\{^{1}H\}$ NMR spectrum of [Be(η^5-Cp)($CH_2P(iPr)_2$)] in C_6D_6.*

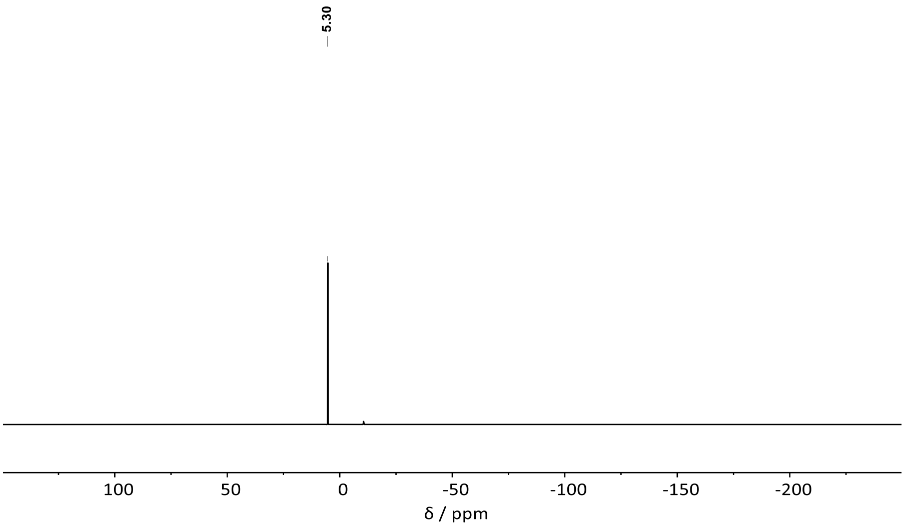

Figure S72. $^{31}P\{^{1}H\}$ NMR spectrum of [Be(η^5-Cp)($CH_2P(iPr)_2$)] in C_6D_6.*

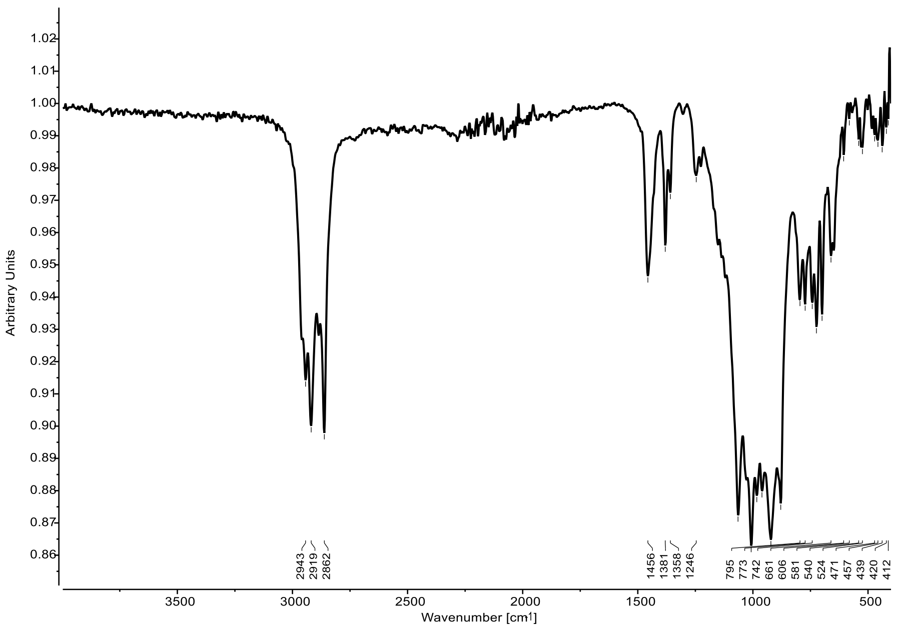

Figure S73. IR spectrum of [Be(η^5-Cp)($CH_2P(iPr)_2$)].*

Reaction with small molecules

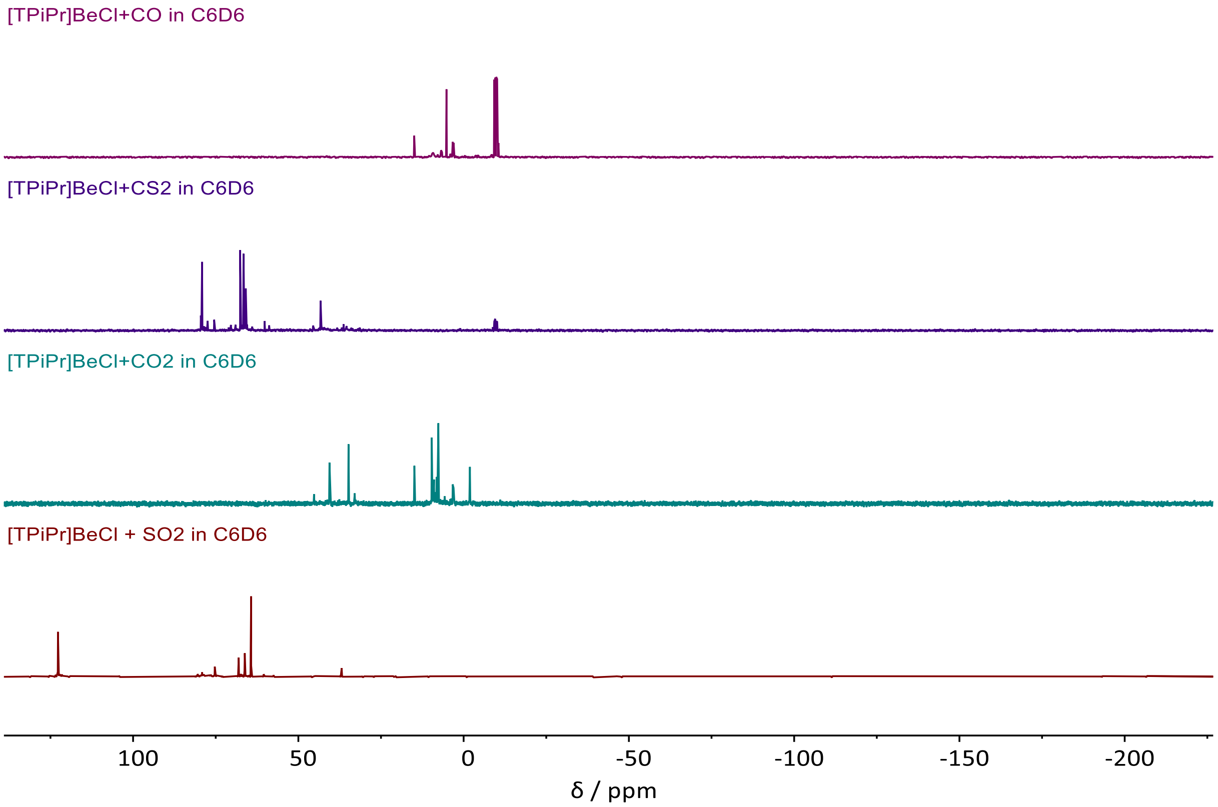

Figure S74. $^{31}P\{^1H\}$ *NMR spectra of [TP(iPr)]BeCl with small molecules in* C_6D_6.

References

[1] a) M. R. Buchner, *Chem. Commun.* **2020**, *56*, 8895–8907; b) M. R. Buchner, *Z. Naturforsch., B: Chem. Sci.* **2020**, *75*, 405–412.

[2] D. Naglav, M. R. Buchner, G. Bendt, F. Kraus, S. Schulz, *Angew. Chem. Int. Ed.* **2016**, *128*, 10718–10733.

[3] M. R. Buchner, M. Müller, *ACS Chem. Health Saf.* **2023**, *30*, 36–43.

[4] T. A. Betley, J. C. Peters, *Inorg. Chem.* **2003**, *42*, 5074–5084.

[5] C. Berthold, G. Hoß, M. H. Lochte, M. R. Buchner, *Inorg. Chem.* **2024**, *63*, 24392–24399.

[6] D. Naglav, B. Tobey, A. Neumann, D. Bläser, C. Wölper, S. Schulz, *Organometallics* **2015**, *34*, 3072–3078.

[7] D. A. Drew, A. Haaland, A. F. Andresen, M. J. Tricker, S. Svensson, *Acta Chem. Scand.* **1972**, *26*, 3351–3356.

[8] M. R. Buchner, L. R. Thomas-Hargreaves, C. Berthold, D. F. Bekiş, S. I. Ivlev, *Chem. Eur. J.* **2023**, *29*, e202302495.

[9] C. Berthold, G. Stebens, B. Butschke, I.-A. Bischoff, A. Schäfer, C. Ding, S. Pan, M. R. Buchner, *Inorg. Chem. Front.* **2024**, DOI: 10.1039/D4QI03234A.

[10] a) G. Brauer (Ed.) *Handbuch der präparativen anorganischen Chemie. 3., umgearb. Aufl.*, Enke, Stuttgart, **1981**; b) A. Kumar, I. Sevonkaev, D. V. Goia, *Journal of Colloid and Interface Science* **2014**, *416*, 119–123.

[11] MestReNova, *Mestrelab Research S.L.*, Santiago de Compostela, Spain, **2011**.

[12] *APEX3 V2019. 11-2*, Bruker AXS Inc., Madison, Wisconsin, USA, **2019**.

[13] *APEX5 V2023. 9-2*, Bruker AXS Inc., Madison, Wisconsin, USA, **2023**.

[14] a) *SADABS/SAINT*, SADABS/SAINT, Madison, Wisconsin, USA, **2016**; b) *SADABS/SAINT within APEX5*, SADABS/SAINT, Madison, Wisconsin, USA, **2018**.

[15] G. M. Sheldrick, *Acta Crystallogr. Sec. A. Found. Adv.* **2015**, *71*, 3–8.

[16] G. M. Sheldrick, *Acta Crystallogr. Sec. C. Struct. Chem.* **2015**, *C71*, 3–8.

[17] O. V. Dolomanov, A. J. Blake, N. R. Champness, M. Schröder, *J. Appl. Crystallogr.* **2003**, *36*, 1283–1284.

[18] K. Brandenburg, H. Putz, *Diamond - Crystal and Molecular Structure Visualization, V 4.6.8*, Crystal Impact GbR, Bonn, **2022**.

[19] *OPUS V7.2*, Bruker Optik GmbH, Ettlingen, Germany, **2012**.
